# Supplementary material for: Gut microbiota suppress feeding induced by palatable foods
Source: Curr Biol. 2023 Jan 9;33(1):147–157.e7. doi: 10.1016/j.cub.2022.10.066 (PMC9839363; doi:10.1016/j.cub.2022.10.066)
Supplement: Document S1. Figures S1–S4 and Table S3 [file mmc1.pdf]

Current Biology, Volume *33*

Supplemental Information

Gut microbiota suppress feeding induced by palatable foods

James Ousey, Joseph C. Boktor, and Sarkis K. Mazmanian

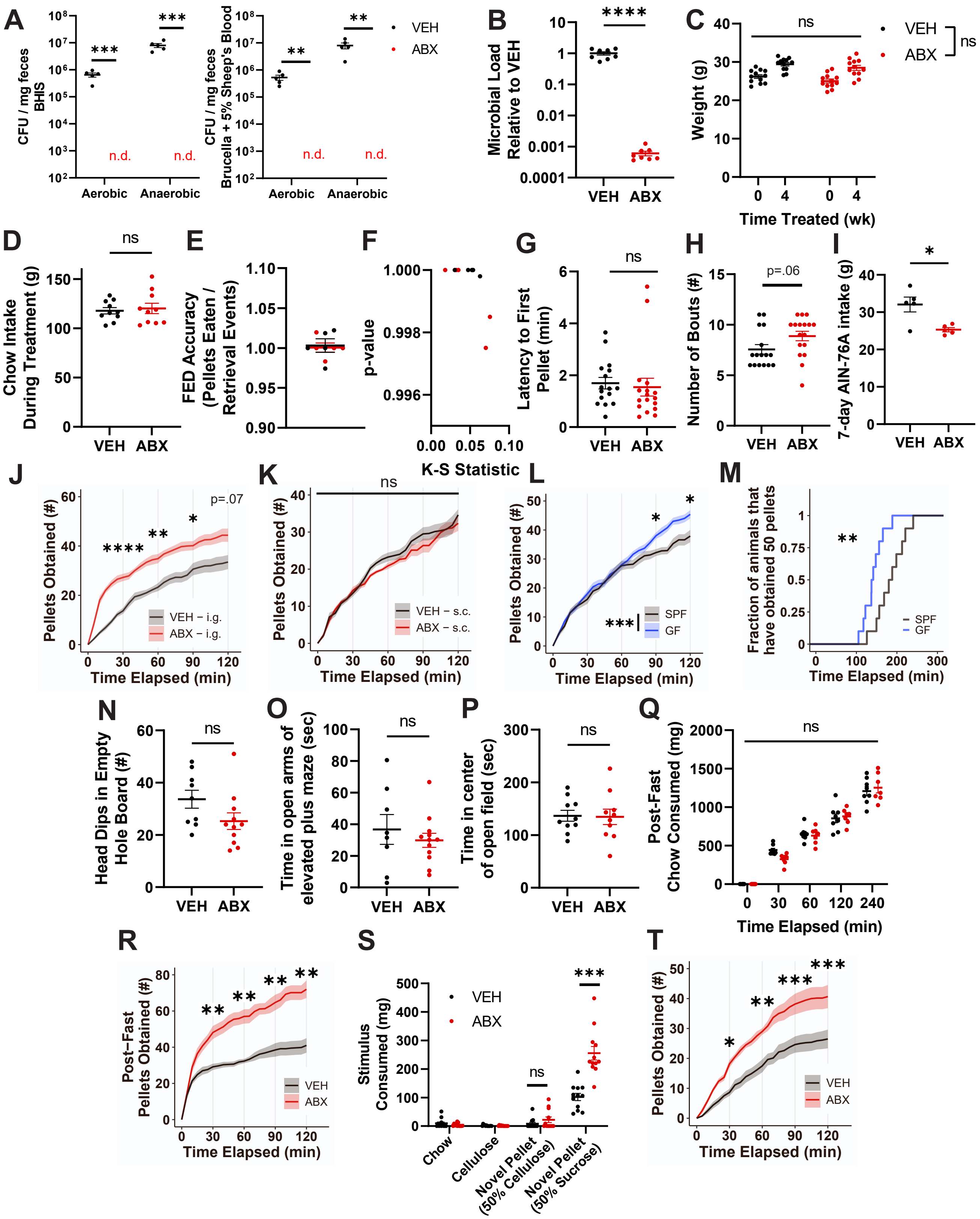
A
CFU / mg feces BHIS
Aerobic
Anaerobic
n.d.
CFU / mg feces Brucella + 5% Sheep's Blood
VEH
ABX
B
Microbial Load Relative to VEH
C
Weight (g)
Time Treated (wk)
D
Chow Intake During Treatment (g)
E
FED Accuracy (Pellets Eaten / Retrieval Events)
F
p-value
K-S Statistic
G
Latency to First Pellet (min)
H
Number of Bouts (#)
p=.06
I
7-day AIN-76A intake (g)
J
Pellets Obtained (#)
VEH – i.g.
ABX – i.g.
Time Elapsed (min)
p=.07
K
VEH – s.c.
ABX – s.c.
L
SPF
GF
M
Fraction of animals that have obtained 50 pellets
N
Head Dips in Empty Hole Board (#)
O
Time in open arms of elevated plus maze (sec)
P
Time in center of open field (sec)
Q
Post-Fast Chow Consumed (mg)
R
Post-Fast Pellets Obtained (#)
S
Stimulus Consumed (mg)
Chow
Cellulose
Novel Pellet (50% Cellulose)
Novel Pellet (50% Sucrose)
T
ns

Figure S1. Physiological and behavioral measurements of microbiota-depleted mice reveal effects on food intake are robust and depend on dietary composition, Related to Figure 1.

(A) Quantification of aerobic and anaerobic microbial growth via colony-forming-unit analysis from fecal samples of VEH and ABX mice (n=5/group) on non-selective media after 4 weeks of treatment. n.d.: not detected. Shown is the mean (± SEM). Significance calculated via two-tailed Student's t-tests.

(B) Microbial load in fecal samples relative to VEH mice as measured by qPCR with universal bacterial primers. Shown is the mean (± SEM). Significance calculated via two-tailed Student's t-test (n=8/group).

(C) Body weight of VEH and ABX-treated animals (n=12/group) immediately prior to and after 4 weeks of treatment. Shown is the mean (± SEM). Treatment effect and Time × Treatment interaction effect significance calculated via two-way repeated measures ANOVA.

(D) Chow intake of single-housed VEH and ABX (n=10/group) mice over 4 weeks of treatment. Shown is the mean (± SEM). Significance calculated via two-tailed Student's t-test.

(E) Quantification of FED reflection of consumption behavior in VEH and ABX mice (n=5/group) determined via comparison of FED-recorded high-sucrose pellet retrieval events to manual video analysis of pellets consumed during 2 hours of free access. Shown is the mean (± SEM) for both treatment groups.

(F) K-S statistics and p-values of within-subject comparisons of cumulative distribution functions of FED-recorded pellet retrieval events and manually recorded pellet consumption events in VEH and ABX mice (n=5/group).

(G) Latency to retrieve the first pellet in VEH (n=16) and ABX (n=17) mice. Shown is the mean (± SEM). Significance calculated via two-tailed Student's t-test.

(H) Cumulative feeding bouts over the first 2 hours of high-sucrose pellet exposure in VEH (n=16) and ABX (n=17) mice. Shown is the mean (± SEM). Significance calculated via two-tailed Student's t-test.

(I) Home cage intake of high-sucrose AIN-76A tablets in single-housed VEH and ABX (n=5/group) mice over 1 week. Shown is the mean (± SEM). Significance calculated via two-tailed Student's t-test.

(J) Cumulative retrieval of high-sucrose pellets between VEH-gavage (VEH – i.g., n=10) and ABX-gavage (ABX – i.g., n=8) mice. Shown is the mean (± SEM) plotted every 5 minutes. Significance calculated via two-way repeated measures ANOVA using 30-minute timepoints followed by Šidák's multiple comparisons test.

(K) Cumulative retrieval of high-sucrose pellets between VEH-subcutaneous (VEH – s.c.) and ABX-subcutaneous (ABX – s.c.) mice (n=8/group). Shown is the mean (± SEM) plotted every 5 minutes. Time × Treatment interaction effect significance calculated via two-way repeated measures ANOVA using 30-minute timepoints.

(L) Cumulative retrieval of high-sucrose pellets between SPF and germ-free (GF) mice (n=10/group). Shown is the mean (± SEM) plotted every 5 minutes. Timepoint and Time × Treatment interaction effect significance calculated via two-way repeated measures ANOVA using 30-minute timepoints followed by Šidák's multiple comparisons test.

(M) Empirical cumulative distribution plot of mice in SPF and GF cohorts that have retrieved 50 high-sucrose pellets (1 gram). Significance calculated via Mann-Whitney U test.

(N) Spontaneous exploratory head dips from VEH (n=9) and ABX (n=11) mice in a 10-minute empty hole-board assay. Shown is the mean (± SEM). Significance calculated via two-tailed Student's t-test.

(O) Open-arm time measurements for VEH (n=8) and ABX (n=12) in an elevated plus maze assay for anxiety. Shown is the mean (± SEM). Significance calculated via two-tailed Student's t-test.

(P) Exposed center time measurements for VEH and ABX (n=10/group) in an open field assay for anxiety. Shown is the mean (± SEM). Significance calculated via two-tailed Student's t-test.
(Q) Fasting-refeeding measurements of fasted VEH (n=8) and ABX (n=7) mice when refed with standard chow. Shown is the mean (± SEM). Time × Treatment interaction effect significance calculated via two-way repeated measures ANOVA.
(R) Fasting-refeeding pellet retrieval measurements of fasted VEH (n=6) and ABX (n=7) mice when refed with high-sucrose pellets. Shown is the mean (± SEM) plotted every 5 minutes. Significance calculated via two-way repeated measures ANOVA using 30-minute timepoints followed by Šidák's multiple comparisons test.
(S) Intake during 10-minute brief-access assays of different edible stimuli presented to VEH and ABX treated mice (n=12/group). Shown is the mean (± SEM). Significance calculated via two-way repeated measures ANOVA followed by Šidák's multiple comparisons test.
(T) Cumulative retrieval of 2% (w/w) sucralose pellets in VEH (n=8) and ABX (n=6) mice. Shown is the mean (± SEM) plotted every 5 minutes. Significance calculated via two-way repeated measures ANOVA using 30-minute timepoints followed by Šidák's multiple comparisons test.
****p<0.0001, ***p<0.001, **p<0.01, *p<0.05, ns: not significant.

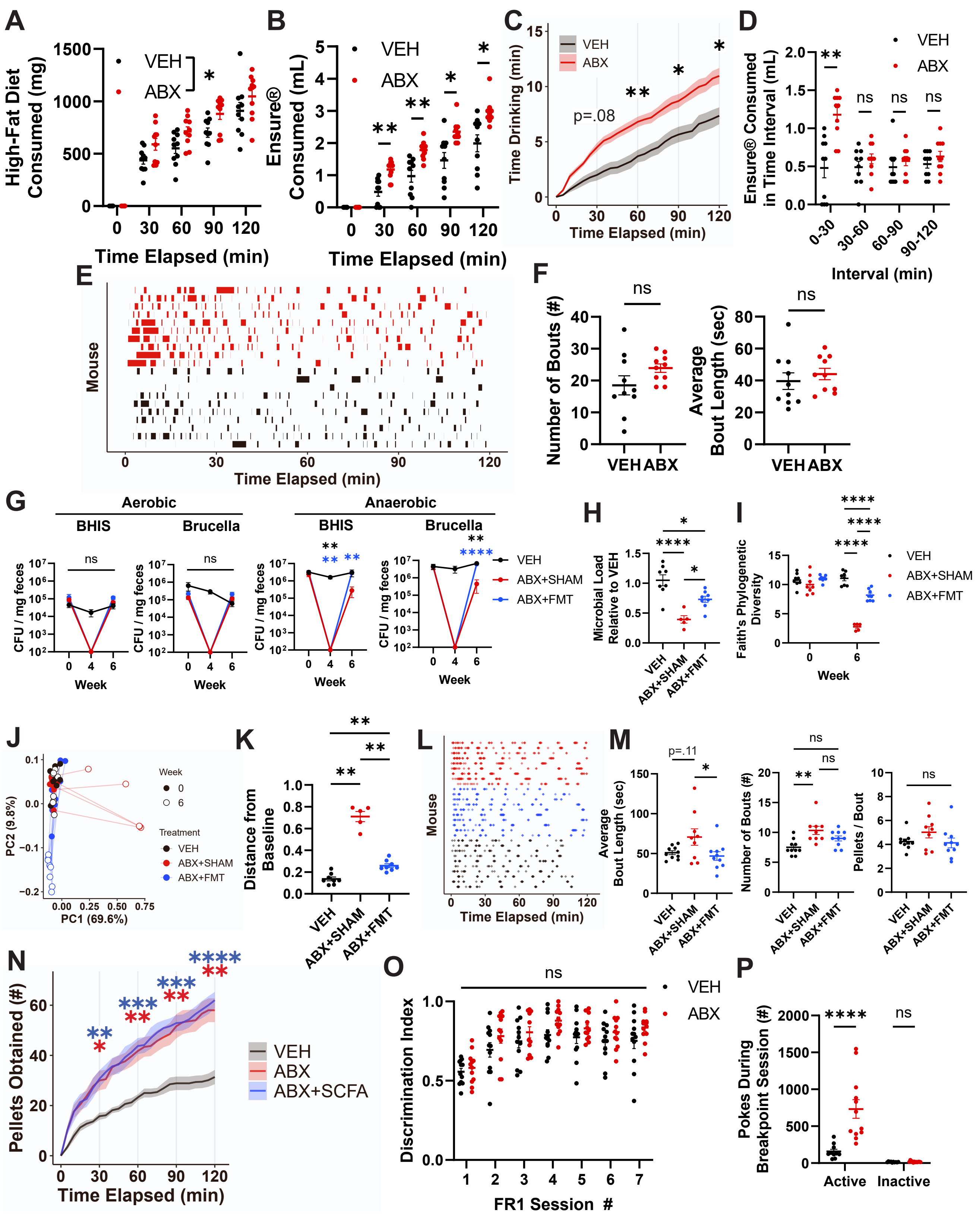

A
B
C
D
E
F
G
H
I
J
K
L
M
N
O
P
High-Fat Diet Consumed (mg)
Ensure® Consumed (mL)
Time Elapsed (min)
Time Drinking (min)
p=.08
Ensure® Consumed in Time Interval (mL)
Interval (min)
VEH
ABX
ns
Mouse
Number of Bouts (#)
Average Bout Length (sec)
Aerobic
Anaerobic
BHIS
Brucella
CFU / mg feces
Week
ABX+SHAM
ABX+FMT
Microbial Load Relative to VEH
Faith's Phylogenetic Diversity
PC1 (69.6%)
PC2 (9.8%)
Treatment
Distance from Baseline
p=.11
Pellets / Bout
Pellets Obtained (#)
ABX+SCFA
Discrimination Index
FR1 Session #
Pokes During Breakpoint Session (#)
Active
Inactive

Figure S2. Additional behavioral parameters from free-feeding and operant conditioning assays and microbiome diversity measurements of FMT recipient mice, Related to Figures 1 and 2

(A) Cumulative consumption of a high-fat diet in VEH and ABX mice (n=10/group). Shown is the mean (± SEM). Treatment effect significance calculated via two-way repeated measures ANOVA at 30-minute timepoints followed by Šidák's multiple comparisons test.

(B) Cumulative consumption of Ensure® in VEH and ABX mice (n=10/group). Shown is the mean (± SEM). Significance calculated via two-way repeated measures ANOVA at 30-minute timepoints followed by Šidák's multiple comparisons test.

(C) Cumulative time drinking Ensure® in VEH and ABX mice (n=10/group). Shown is the mean (± SEM). Significance calculated via two-way repeated measures ANOVA at 30-minute timepoints followed by Šidák's multiple comparisons test.

(D) Consumption of Ensure® in VEH and ABX mice (n=10/group) across 30-minute intervals. Shown is the mean (± SEM). Significance calculated via two-way repeated measures ANOVA followed by Šidák's multiple comparisons test.

(E) Raster plot of Ensure® drinking bouts in VEH and ABX mice.

(F) Bout structure analyses of Ensure ® drinking bouts over 2 hours of access. Shown is the mean (± SEM). Significance calculated via two-tailed Student's t-test.

(G) Quantification of aerobic and anaerobic microbial growth via colony-forming-unit analysis from fecal samples of VEH, ABX+SHAM, and ABX+FMT mice ((Week 6, ABX+SHAM n=5), n=8/group all others) on non-selective media over the course of treatment. Shown is the mean (± SEM). Significance calculated via mixed-effects modeling followed by Tukey's multiple comparisons test (within timepoints). Black and blue asterisks denote ABX+SHAM vs. VEH and ABX+SHAM vs. ABX+FMT comparison significance, respectively.

(H) Microbial load in fecal samples of ABX+SHAM (n=5) and ABX+FMT (n=8) mice relative to VEH (n=8) mice at 6 weeks of treatment (2 weeks post-FMT) as measured by qPCR with universal bacterial primers. Shown is the mean (± SEM). Significance calculated via one-way ANOVA followed by Tukey's multiple comparisons test.

(I) Faith's Phylogenetic Diversity in VEH (n=8), ABX+SHAM (Week 0 n=8, Week 6 n=5), and ABX+FMT (n=8) mice at baseline and 6 weeks of treatment. Shown is the mean (± SEM). Significance calculated via mixed-effects modeling followed by Tukey's multiple comparisons test (within timepoints).

(J) PCoA of weighted UniFrac distances in VEH (n=8), ABX+SHAM (Week 0 n=8, Week 6 n=5), and ABX+FMT (n=8) mice at baseline and at 6 weeks of treatment.

(K) Within-subject weighted UniFrac distances in VEH (n=8), ABX+SHAM (n=5), and ABX+FMT (n=8) mice at 6 weeks of treatment compared to baseline. Shown is the mean (± SEM). Significance calculated via one-way ANOVA Kruskal-Wallis of within-subject distance comparisons to baseline followed by Mann-Whitney U tests subject to FDR correction as implemented in QIIME2. See also Table S1.

(L) Raster plot of pellet retrieval events of mice in VEH, ABX+SHAM, and ABX+FMT cohorts.

(M) Microstructure analyses of pellet retrieval events of mice in VEH, ABX+SHAM, and ABX+FMT cohorts over the first 2 hours of high-sucrose pellet exposure. Shown is the mean (± SEM). Significance calculated via one-way ANOVA followed by Tukey's multiple comparisons test.

(N) Cumulative retrieval of high-sucrose pellets of VEH, ABX, and ABX+SCFA (n=8/group) mice. Shown is the mean (± SEM) plotted every 5 minutes. Significance calculated via two-way repeated measures ANOVA using 30-minute timepoints followed by Tukey's multiple comparisons test (within timepoints). Red and blue asterisks denote ABX vs. VEH and ABX+SCFA vs. VEH comparison significance, respectively.

(O) Discrimination Indices (active pokes/total pokes) between VEH and ABX (n=12/group) mice during the first 7 days of FR1 training sessions. Shown is the mean (± SEM). Session × Treatment interaction effect significance calculated via two-way repeated measures ANOVA.
(P) Total active and inactive pokes in VEH (n=10) and ABX (n=12) mice during the progressive ratio breakpoint session. Shown is the mean (± SEM). Significance calculated via two-way repeated measures ANOVA followed by Šidák’s multiple comparisons test.

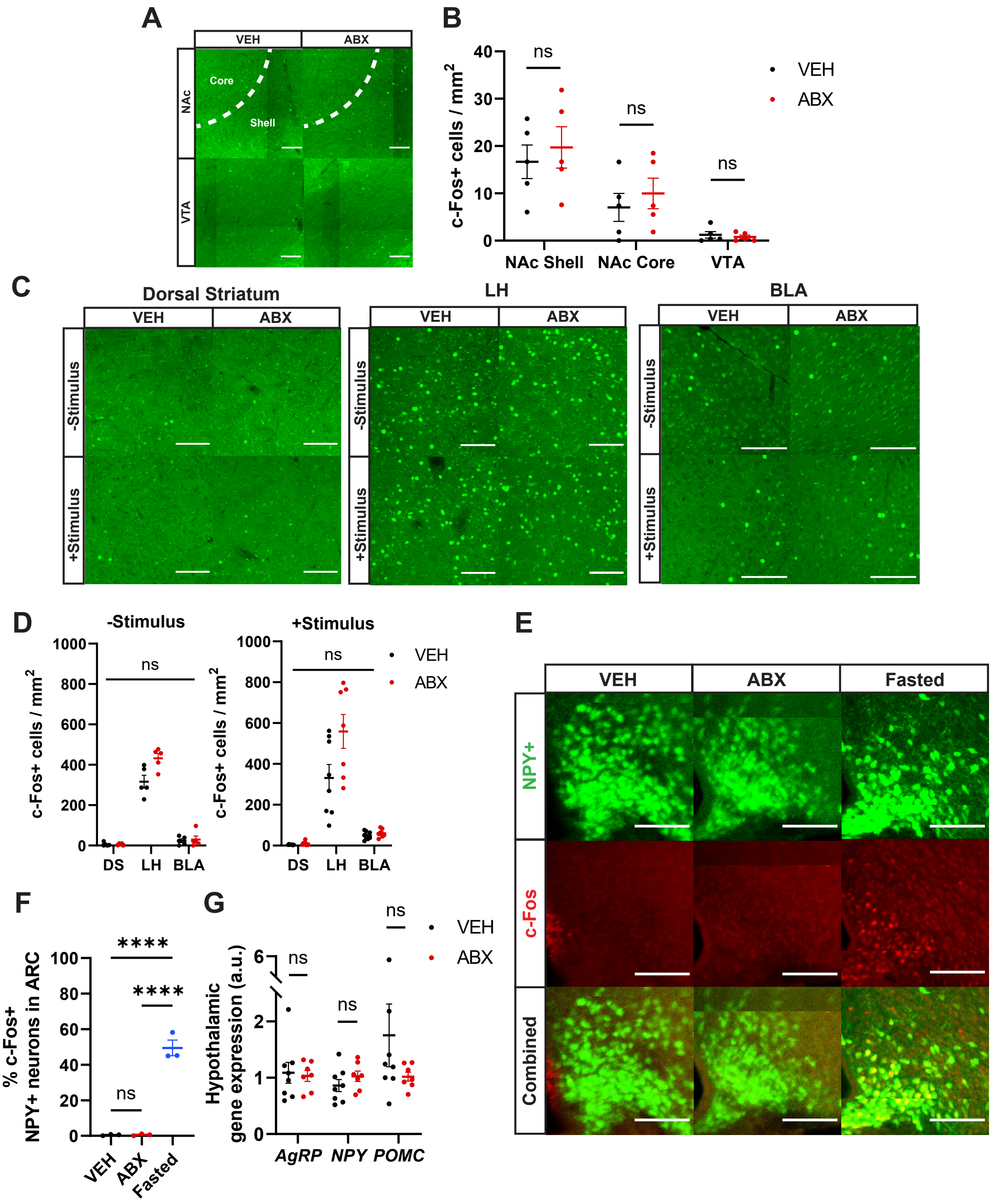

A
VEH
ABX
NAc
Core
Shell
VTA
B
c-Fos+ cells / mm2
ns
VEH
ABX
NAc Shell
NAc Core
VTA
C
Dorsal Striatum
LH
BLA
-Stimulus
+Stimulus
D
-Stimulus
+Stimulus
c-Fos+ cells / mm2
DS
LH
BLA
E
VEH
ABX
Fasted
NPY+
c-Fos
Combined
F
% c-Fos+ NPY+ neurons in ARC
****
ns
VEH
ABX
Fasted
G
Hypothalamic gene expression (a.u.)
AgRP
NPY
POMC

Figure S3. High-sucrose pellet-induced neural activity in reward-related brain regions and characterization of homeostatic feeding signals in VEH and ABX mice, Related to Figure 2

(A) Representative images of the nucleus accumbens (NAc) and ventral tegmental area (VTA) in VEH and ABX mice without access to high-sucrose pellets, with c-Fos intensity represented in green. Scale bar is 100 microns. Images are cropped to emphasize the region of interest.

(B) Density of c-Fos+ neurons in the NAc shell, NAc core, and VTA (n=5/group) without access to high-sucrose pellets. Shown is the mean (± SEM). Significance calculated via two-way ANOVA with microbiota status and access to high-sucrose pellets as factors, followed by Bonferroni's multiple comparisons test (within brain regions). Data for mice given access to high-sucrose pellets is shown in Figures 2D and 2E.

(C) Representative images of c-Fos+ neurons in the dorsal striatum, lateral hypothalamus (LH), and basolateral amygdala (BLA) with (+Stimulus) and without (-Stimulus) 1 hour of access to high-sucrose pellets. Images have been cropped to aid cell body visualization. Scale bar is 100 microns.

(D) Density of c-Fos+ neurons in the dorsal striatum, LH, and BLA with (dorsal striatum: n=8/group, LH and BLA: n=8 VEH, n=7 ABX) and without (all regions: n=5/group) 1 hour of access to high-sucrose pellets. Shown is the mean (± SEM). Within-brain region Treatment × Stimulus interaction significance calculated via two-way ANOVA with microbiota status and access to high-sucrose pellets as factors.

(E–F) Representative images (E) and quantification (F) of c-Fos+ NPY+ neurons in the arcuate nucleus of the hypothalamus of VEH, ABX, or fasted (n=3/group) *NPY-hrGFP* transgenic mice. Shown is the mean (± SEM). Significance calculated via one-way ANOVA followed by Tukey's multiple comparisons test. Images have been cropped to aid cell body visualization. Scale bar is 100 microns.

(G) Hypothalamic neuropeptide expression as measured by qPCR in VEH (n=8) and ABX (n=7) mice. Shown is the mean (± SEM). Significance calculated via two-tailed Student's t-test followed by Holm-Šidák's correction for multiple comparisons.

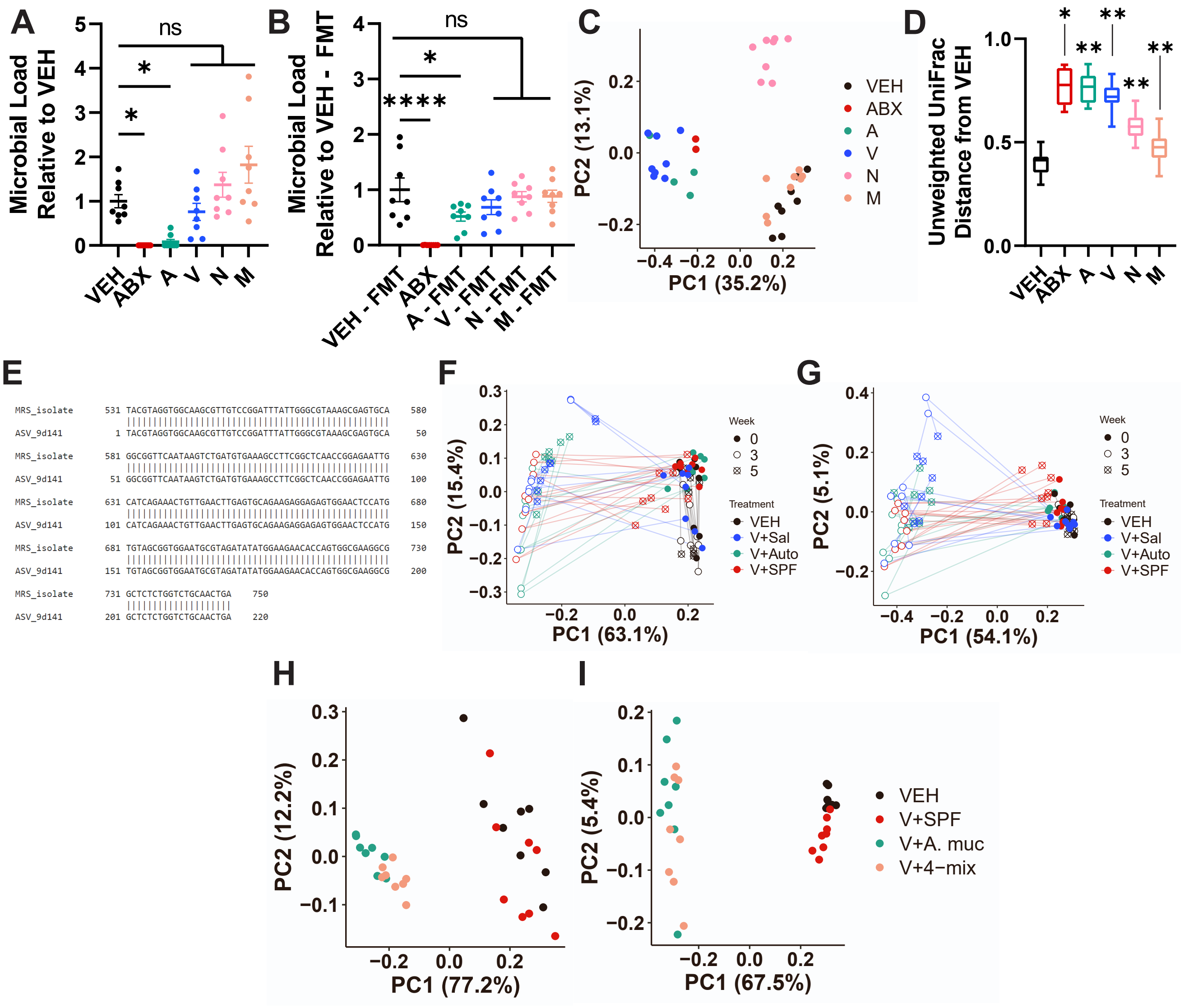
A
Microbial Load Relative to VEH
ns
*
*
VEH
ABX
A
V
N
M
B
Microbial Load Relative to VEH - FMT
ns
*
****
VEH - FMT
ABX
A - FMT
V - FMT
N - FMT
M - FMT
C
PC2 (13.1%)
PC1 (35.2%)
VEH
ABX
A
V
N
M
D
Unweighted UniFrac Distance from VEH
*
**
**
**
**
E
MRS_isolate 531 TACGTAGGTGGCAAGCGTTGTCCGGATTTATTGGGCGTAAAGCGAGTGCA 580
ASV_9d141 1 TACGTAGGTGGCAAGCGTTGTCCGGATTTATTGGGCGTAAAGCGAGTGCA 50
MRS_isolate 581 GGCGGTTCAATAAGTCTGATGTGAAAGCCTTCGGCTCAACCGGAGAATTG 630
ASV_9d141 51 GGCGGTTCAATAAGTCTGATGTGAAAGCCTTCGGCTCAACCGGAGAATTG 100
MRS_isolate 631 CATCAGAAACTGTTGAACTTGAGTGCAGAAGAGGAGAGTGGAACTCCATG 680
ASV_9d141 101 CATCAGAAACTGTTGAACTTGAGTGCAGAAGAGGAGAGTGGAACTCCATG 150
MRS_isolate 681 TGTAGCGGTGGAATGCGTAGATATATGGAAGAACACCAGTGGCGAAGGCG 730
ASV_9d141 151 TGTAGCGGTGGAATGCGTAGATATATGGAAGAACACCAGTGGCGAAGGCG 200
MRS_isolate 731 GCTCTCTGGTCTGCAACTGA 750
ASV_9d141 201 GCTCTCTGGTCTGCAACTGA 220
F
PC2 (15.4%)
PC1 (63.1%)
Week
0
3
5
Treatment
VEH
V+Sal
V+Auto
V+SPF
G
PC2 (5.1%)
PC1 (54.1%)
Week
0
3
5
Treatment
VEH
V+Sal
V+Auto
V+SPF
H
PC2 (12.2%)
PC1 (77.2%)
I
PC2 (5.4%)
PC1 (67.5%)
VEH
V+SPF
V+A. muc
V+4−mix

Figure S4. Differential antibiotic administration and microbial treatment has distinct effects on microbial abundance and diversity, Related to Figures 3 and 4

(A) Microbial load in fecal samples relative to VEH mice (n=8/group) as calculated by qPCR with universal bacterial primers. Shown is the mean (± SEM). Significance calculated via one-way ANOVA followed by Dunnett's multiple comparisons test to VEH.

(B) Microbial load in fecal samples relative to VEH - FMT mice (n=8/group) as calculated by qPCR with universal bacterial primers. Shown is the mean (± SEM). Significance calculated via one-way ANOVA followed by Dunnett's multiple comparisons test to VEH - FMT.

(C–D) PCoA and boxplot of pairwise comparisons of unweighted UniFrac distances in VEH (n=8), ABX (n=2), A (n=4), V (n=8), N (n=8), M (n=8) animals. Significance calculated by PERMANOVA. Asterisks in (D) denote significance of the PERMANOVA test of treatment groups compared to VEH. See also Table S1.

(E) EMBOSS matcher alignment of the 16S rRNA DNA sequence of a *L. johnsonii* isolate cultured from SPF feces (top) to the significantly differentially abundant ASV aligning to *Lactobacillus* sp. (bottom).

(F–G) PCoA of weighted (F) and unweighted (G) UniFrac distances of fecal microbiomes from vehicle (VEH) or vancomycin-pretreated mice that received treatment with saline vehicle (V+Sal), autologous FMT (V+Auto), or FMT from an SPF donor (V+SPF). Significance calculated via within-timepoint one-way ANOVA Kruskal-Wallis of within-subject distance comparisons to baseline followed by Mann-Whitney U tests subject to FDR correction as implemented in QIIME2 (n=8/group). See also Table S1.

(H–I) PCoA of weighted (H) and unweighted (I) UniFrac distances of fecal microbiomes from VEH, V+SPF, V+*A. muc*, and V+4-mix (n=8/group) mice. Significance calculated via PERMANOVA. See also Table S1.

Gene	Forward/Reverse	Source	Sequence (5'-3')
AgRP	Forward	[S1]	TGCTACTGCCGCTTCTTCAA
AgRP	Reverse	[S1]	CTTTGCCCAAACAACATCCA
NPY	Forward	[S1]	TAACAAGCGAATGGGGCTGT
NPY	Reverse	[S1]	ATCTGGCCATGTCCTCTGCT
POMC	Forward	[S1]	AGGCCTGACACGTGGAAGAT
POMC	Reverse	[S1]	AGGCACCAGCTCCACACAT
18S *rRNA*	Forward	[S2]	TTCCGATAACGAACGAGACTCT
18S *rRNA*	Reverse	[S2]	TGGCTGAACGCCACTTGTC
16S 515F	Forward	[S3]	GTGCCAGCMGCCGCGGTAA
16S 806R	Reverse	[S3]	GGACTACHVGGGTWTCTAAT
16S 27F	Forward	[S4]	AGAGTTTGATCMTGGCTCAG
16S 1492R	Reverse	[S5]	GGTTACCTTGTTACGACTT

Table S3. Oligonucleotides used in this study, Related to STAR Methods

Supplemental References

S1. Piper, M.L., Unger, E.K., Myers, M.G., and Xu, A.W. (2008). Specific Physiological Roles for Signal Transducer and Activator of Transcription 3 in Leptin Receptor-Expressing Neurons. Mol. Endocrinol. *22*, 751–759. 10.1210/me.2007-0389.

S2. Reichenbach, A., Mequinion, M., Bayliss, J.A., Lockie, S.H., Lemus, M.B., Mynatt, R.L., Stark, R., and Andrews, Z.B. (2018). Carnitine Acetyltransferase in AgRP Neurons Is Required for the Homeostatic Adaptation to Restricted Feeding in Male Mice. Endocrinology *159*, 2473–2483. 10.1210/en.2018-00131.

S3. Caporaso, J.G., Lauber, C.L., Walters, W.A., Berg-Lyons, D., Lozupone, C.A., Turnbaugh, P.J., Fierer, N., and Knight, R. (2011). Global patterns of 16S rRNA diversity at a depth of millions of sequences per sample. Proc. Natl. Acad. Sci. *108*, 4516–4522. 10.1073/pnas.1000080107.

S4. Lane, D.J. (1991). 16S/23S rRNA sequencing. In Nucleic acid techniques in bacterial systematics., E. Stackebrandt and M. Goodfellow, eds. (John Wiley and Sons), pp. 115–175.

S5. Turner, S., Pryer, K.M., Miao, V.P., and Palmer, J.D. (1999). Investigating deep phylogenetic relationships among cyanobacteria and plastids by small subunit rRNA sequence analysis. J. Eukaryot. Microbiol. *46*, 327–338. 10.1111/j.1550-7408.1999.tb04612.x.
