# Supplementary material for: Gut microbiota suppress feeding induced by palatable foods
Source: Curr Biol. 2023 Jan 9;33(1):147–157.e7. doi: 10.1016/j.cub.2022.10.066 (PMC9839363; doi:10.1016/j.cub.2022.10.066)
Supplement: Document S2. Article plus supplemental information [file mmc4.pdf]

Current Biology

Report

Gut microbiota suppress feeding induced by palatable foods

Highlights

- Gut microbiota depletion of mice reversibly results in palatable food overconsumption
- Microbiota-depleted mice exhibit greater motivation to pursue a high-sucrose reward
- Colonization with S24-7 and *L. johnsonii* reduces vancomycin-induced binge eating

Authors

James Ousey, Joseph C. Boktor, Sarkis K. Mazmanian

Correspondence

jousey@caltech.edu (J.O.),
sarkis@caltech.edu (S.K.M.)

In brief

Ousey et al. find that gut microbiota depletion in mice results in exacerbated binge-like intake of palatable foods and increased motivation to pursue a high-sucrose reward. They identify microbial taxa correlated with feeding suppression and a mixture of commensal microbes capable of suppressing palatable food intake in vancomycin-treated mice.

Ousey et al., 2023, Current Biology *33*, 147–157
January 9, 2023 © 2022 The Author(s). Published by Elsevier Inc.
https://doi.org/10.1016/j.cub.2022.10.066

Current Biology

CellPress
OPEN ACCESS

Report

Gut microbiota suppress feeding induced by palatable foods

James Ousey,[1,*] Joseph C. Boktor,[1] and Sarkis K. Mazmanian[1,2,*]
[1]Division of Biology and Biological Engineering, California Institute of Technology, 1200 E California Blvd, Pasadena, CA 91125, USA
[2]Lead contact
*Correspondence: jousey@caltech.edu (J. O.), sarkis@caltech.edu (S.K. M.)
https://doi.org/10.1016/j.cub.2022.10.066

SUMMARY

Feeding behaviors depend on intrinsic and extrinsic factors including genetics, food palatability, and the environment.[1–5] The gut microbiota is a major environmental contributor to host physiology and impacts feeding behavior.[6–12] Here, we explored the hypothesis that gut bacteria influence behavioral responses to palatable foods and reveal that antibiotic depletion (ABX) of the gut microbiota in mice results in overconsumption of several palatable foods with conserved effects on feeding dynamics. Gut microbiota restoration via fecal transplant into ABX mice is sufficient to rescue overconsumption of high-sucrose pellets. Operant conditioning tests found that ABX mice exhibit intensified motivation to pursue high-sucrose rewards. Accordingly, neuronal activity in mesolimbic brain regions, which have been linked with motivation and reward-seeking behavior,[3] was elevated in ABX mice after consumption of high-sucrose pellets. Differential antibiotic treatment and functional microbiota transplants identified specific gut bacterial taxa from the family S24-7 and the genus *Lactobacillus* whose abundances associate with suppression of high-sucrose pellet consumption. Indeed, colonization of mice with S24-7 and *Lactobacillus johnsonii* was sufficient to reduce overconsumption of high-sucrose pellets in an antibiotic-induced model of binge eating. These results demonstrate that extrinsic influences from the gut microbiota can suppress the behavioral response toward palatable foods in mice.

RESULTS

Regulation of feeding behaviors is critical for health.[13]Circulating metabolic signals,[1–3] gastrointestinal (GI) feedback,[4,5] and food palatability[14] are integrated to coordinate food pursuit and consumption. In mammals, feeding behavior is subdivided into homeostatic feeding, necessary to maintain energy balance, and hedonic feeding, driven by pleasure.[2,3,14–16]

Hedonic feeding is influenced by food palatability, an ascribed valuation of food reward influenced by taste and past food-associated experiences.[16,17] Under conditions of limited access, palatable food exposure will promptly induce feeding in rodents, even when unfasted.[18,19] This “binge-like” consumption behavior is frequently observed upon access to high-sugar or high-fat foods.[20] Behavioral analyses have uncovered that temporal characteristics of intake, such as feeding bout duration and consumption rate, associate with the sensory pleasure of the diet.[21–25] Operant conditioning assays measure the incentive salience of a palatable food by tracking the effort exerted to obtain a food reward.[26–28] The neural circuitry underlying hedonic feeding, primarily residing within the mesolimbic system, appears distinct from the hypothalamic circuitry regulating homeostatic feeding.[3,29]

The gut microbiota affects host metabolism and expression of feeding behaviors.[6–12] Germ-free (GF) mice and mice whose gut bacterial communities have been depleted with antibiotics show changes in glycemia and levels of circulating feeding hormones.[7,8] Administration of short-chain fatty acids (SCFAs), byproducts of gut microbial fermentation, has anorectic effects in mice.[30,31] Studies have suggested microbiome-mediated effects on homeostatic feeding, but these observations vary depending on diet and age of mice.[7,8,32–34] Recent findings have uncovered gut microbiota influences on host diet selection and that hypothalamic sensing of microbial peptides regulates appetite in mice.[11,12] Regarding hedonic feeding, GF mice consume greater amounts of a sucrose solution over a 2-day period than conventional controls at high (8%–16%), but not low (0.5%–4%), sucrose concentrations, thus demonstrating a palatability-dependent gut microbiota effect on intake behavior.[35] Additionally, fecal microbiota transplantation (FMT) from diet-induced obese (DIO) mice transfers DIO-associated reductions in binge-like consumption to recipient animals.[9] A thorough characterization of gut microbiota effects on palatability-induced food intake behaviors has yet to be reported. Here, we explore how gut microbiota depletion may regulate the intake dynamics and incentive salience of palatable foods and investigate if specific gut bacterial species mediate microbiota-dependent changes in host feeding behavior.

Gut microbiota suppress high-sucrose pellet consumption in mice

To uncover microbiota-dependent differences in response to a palatable food, we treated C57BL/6J specific-pathogen-free

 © 2022 The Author(s). Published by Elsevier Inc.
This is an open access article under the CC BY license (http://creativecommons.org/licenses/by/4.0/).

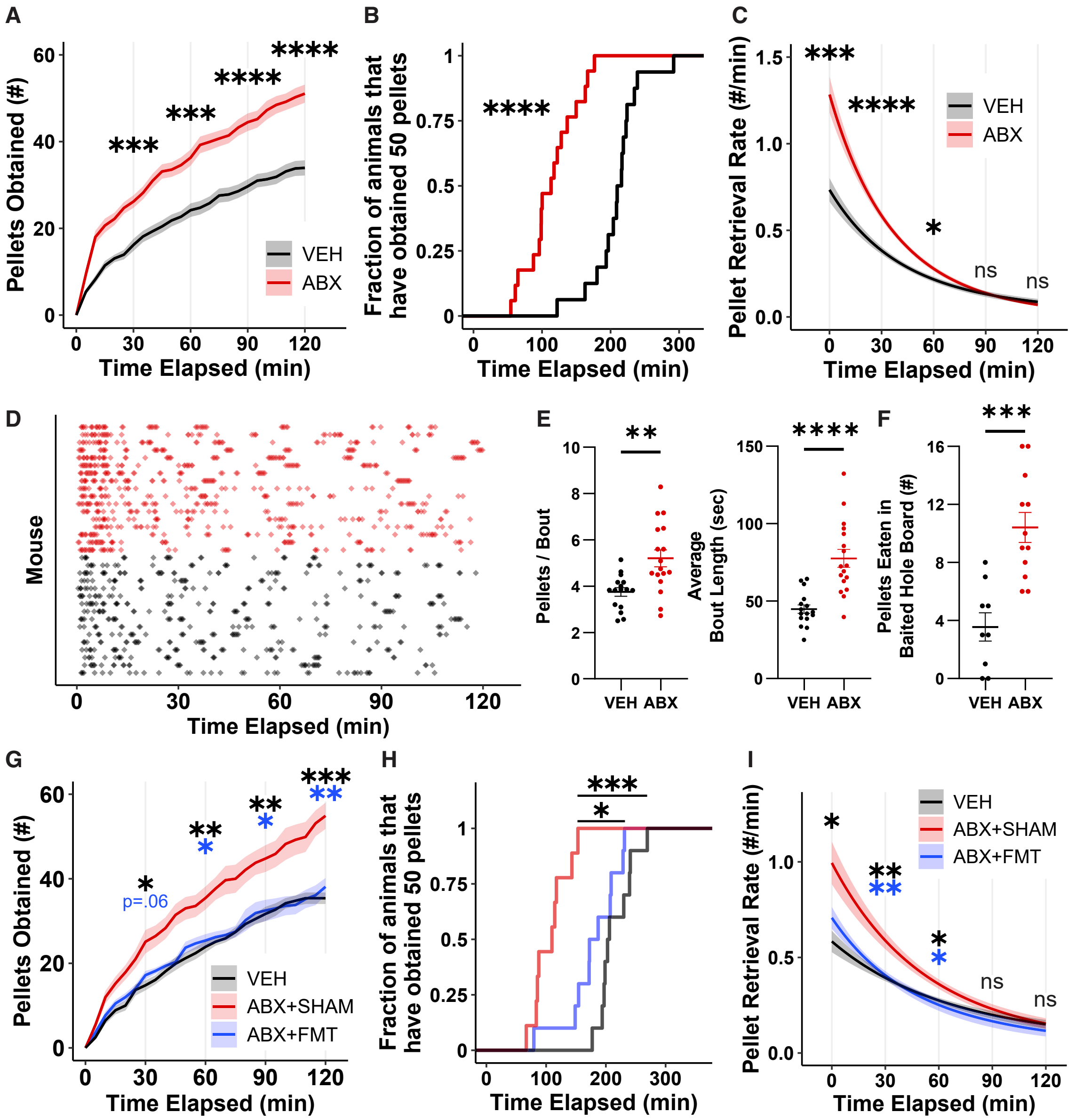

Figure 1. Gut bacteria suppress consumption of high-sucrose pellets in mice

(A) Cumulative retrieval of high-sucrose pellets between VEH (n = 16) and ABX (n = 17) mice. Shown is the mean (± SEM) plotted every 5 min. Significance calculated via two-way repeated measures ANOVA using 30-min time points followed by Šidák's multiple comparisons test.

(B) Empirical cumulative distribution plot of mice in VEH and ABX cohorts that have retrieved 50 high-sucrose pellets (1 g). Significance calculated via Mann-Whitney U test.

(C) Modeled rates of high-sucrose pellet retrieval events for VEH and ABX mice. Shown is the mean (± SEM). Significance calculated via two-way repeated measures ANOVA using 30-min time points followed by Šidák's multiple comparisons test.

(D) Raster plot of pellet retrieval events.

(E) Bout structure analyses of high-sucrose pellet retrieval events over 2 h of access. Shown is the mean (± SEM). Significance calculated via two-tailed Student's t test.

(F) High-sucrose pellets eaten by VEH (n = 9) and ABX (n = 12) mice in a 10-min stimulus-baited hole-board assay. Shown is the mean (± SEM). Significance calculated via two-tailed Student's t test.

(G) Cumulative retrieval of high-sucrose pellets between VEH (n = 10), ABX + SHAM (n = 9), and ABX + FMT (n = 10) mice. Shown is the mean (± SEM) plotted every 5 min. Significance calculated via two-way repeated measures ANOVA using 30-min time points followed by Tukey's multiple comparisons test (within time points). Black and blue asterisks denote ABX + SHAM versus VEH and ABX + SHAM versus ABX + FMT comparison significance, respectively.

(H) Empirical cumulative distribution plot of mice in VEH, ABX + SHAM, and ABX + FMT cohorts that have retrieved 1 g of high-sucrose pellets. Significance calculated via one-way ANOVA Kruskal-Wallis followed by Dunn's multiple comparisons test.

(legend continued on next page)

(SPF) mice with an oral antibiotic mixture that resulted in near-complete depletion of the intestinal commensal microbiota[36–38] without durable weight loss[8,38–40] or changes in homeostatic food intake[8,41] (Figures S1A–S1D). Unfasted antibiotic (ABX) and vehicle (VEH)-treated mice were then given free access to high-sucrose pellets via dispensers which record the times at which pellets are retrieved from the delivery port.[42] We validated that pellet retrieval events reflect consumption behavior via manual scoring of video recordings (Figures S1E and S1F). ABX mice promptly retrieved greater numbers of high-sucrose pellets than VEH mice, with differences in cumulative pellet retrieval persisting for at least 2 h (Figure 1A). After 3 h of high-sucrose pellet availability, 100% (17/17) of the ABX cohort had obtained at least 50 pellets (1 gram) compared with 13% (2/16) of the VEH mice (Figure 1B). Differences in the pellet retrieval rate between ABX and VEH mice were greatest immediately after diet presentation and normalized after 1 h (Figure 1C), a feature not ascribable to differences in latency to retrieve the first pellet (Figure S1G).

Feeding behavior in rodents is characterized by discrete bursts, or "bouts," of intake.[21,43,44] Upon high-sucrose pellet access, feeding bouts of ABX mice were significantly longer than those of VEH mice, with more pellets retrieved per bout (Figures 1D and 1E). ABX mice also demonstrated a strong trend toward an increased number of feeding bouts (Figure S1H). ABX mice exclusively fed the same high-sucrose diet *ad libitum* consumed less than VEH controls (Figure S1I). We controlled for VEH and possible off-target antibiotic effects by administering ABX via intragastric[8] or subcutaneous routes (Figures S1J and S1K). Additionally, GF mice overconsumed high-sucrose pellets compared with SPF controls, although significant between-group differences manifested only after more than 1 h had passed (Figures S1L and S1M).

ABX mice consumed significantly more high-sucrose pellets than VEH mice in a hole-board arena despite exhibiting similar levels of exploratory behavior, suggesting novelty-induced hypophagia associated with the food dispenser did not drive differences in pellet consumption[45,46] (Figures 1F and S1N). Furthermore, VEH and ABX mice did not display differences in generalized anxiety, a potential contributor to hyponeophagia,[47] as measured by the elevated plus maze and open field assays[48–51] (Figures S1O–S1P). Our data demonstrate that the absence of a gut microbiota in mice results in high-sucrose pellet overconsumption.

Gut microbiota reduce intake of various palatable foods

To evaluate if ABX mice universally overconsume in states of excessive intake, perhaps due to reduced post-ingestive negative feedback,[52,53] we induced hyperphagia by fasting mice and refeeding with standard chow or high-sucrose pellets. There was no effect of microbiota depletion in mice refed with chow (Figure S1Q). By contrast, ABX mice refed with high-sucrose pellets consumed approximately 60% more than VEH mice within 2 h (Figure S1R), suggesting that microbiota effects on hyperphagic behavior depend on dietary composition. Next, VEH and ABX mice were given access to high-sucrose pellets or a mimic containing taste-inert cellulose.[54] Only the sucrose-containing pellets induced differential consumption between groups (Figure S1S). Remarkably, ABX mice also overconsumed pellets containing the non-metabolizable sweetener sucralose compared with VEH controls, suggesting the energy provided by dietary sucrose was unnecessary for microbiota-dependent intake differences (Figure S1T).

We tested if microbiota intake suppression extended to other palatable foods reported to prompt binge-like consumption in mice.[55,56] Gut microbiota depletion significantly augmented consumption of a high-fat diet (HFD) (Figure S2A) and Ensure (Figures S2B and S2C). In agreement with our high-sucrose pellet observations, the differences in Ensure intake rate were greatest at the beginning of food access (Figure S2D). However, significant effects on the number and duration of Ensure drinking bouts were not observed (Figures S2E and S2F). Thus, microbiota depletion increases spontaneous feeding of various palatable foods.

Microbiota restoration reverses high-sucrose pellet overconsumption of ABX mice

The gut microbiota of ABX animals can be restored through FMT.[57] We treated ABX mice with fecal transplants from SPF donors (ABX + FMT) or saline (ABX + SHAM) and confirmed that after 2 weeks, ABX + FMT mice had greater fecal microbial load, increased gut microbiome diversity, and harbored gut communities phylogenetically more similar to their pre-ABX state than ABX + SHAM mice (Figures S2G–S2K). ABX + FMT mice retrieved fewer high-sucrose pellets than ABX + SHAM mice, with cumulative intake not significantly differing from VEH mice (Figures 1G and 1H). The pellet retrieval dynamics of ABX + FMT mice recapitulated those of VEH mice, notably with a blunted response in the first hour after high-sucrose pellet presentation compared with ABX + SHAM treatment (Figures 1I and S2L). Furthermore, gut microbiota restoration was sufficient to rescue the increase in average bout length (Figure S2M). These findings are unlikely to depend on microbially produced SCFAs, as SCFA supplementation of ABX mice had no effect on high-sucrose pellet retrieval (Figure S2N). Collectively, a complex gut microbiota is sufficient to suppress feeding induced by a high-sucrose diet in mice.

Gut microbiota alter the incentive salience of a palatable reward

To test if gut microbiota regulate the incentive salience of a high-sucrose reward, we trained VEH and ABX mice in a nose-poke operant conditioning paradigm[58] (Figure 2A). During 1-h fixed-ratio 1 (FR1) training sessions, ABX mice retrieved approximately 50% more high-sucrose pellets each day than VEH mice (Figure 2B), with no between-treatment differences in learning

(I) Modeled rates of pellet retrieval events for VEH, ABX + SHAM, and ABX + FMT mice. Shown is the mean (± SEM). Significance calculated via two-way repeated measures ANOVA using 30-min time points followed by Tukey's multiple comparisons test (within time points). Black and blue asterisks denote ABX + SHAM versus VEH and ABX + SHAM versus ABX + FMT comparison significance, respectively.

****$p < 0.0001$, ***$p < 0.001$, **$p < 0.01$, *$p < 0.05$, ns, not significant.

See also Figures S1 and S2.

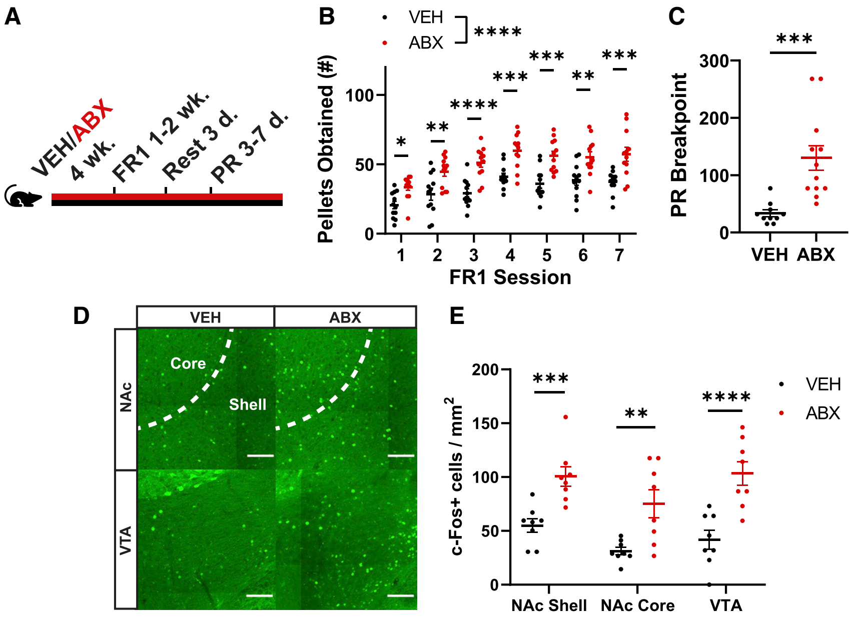

Figure 2. Gut microbiota reduce the incentive salience of a high-sucrose reward and decrease activity in mesolimbic brain regions linked to reward behaviors

(A) Schematic illustrating timeline of fixed-ratio 1 (FR1) training and progressive ratio (PR) breakpoint testing.

(B) High-sucrose pellets obtained during daily FR1 training sessions of VEH (n = 12) and ABX (n = 12) mice. Shown is the mean (± SEM). Significance calculated via two-way repeated measures ANOVA followed by Šidák's multiple comparisons test.

(C) Breakpoints of VEH (n = 10) and ABX (n = 12) mice from the progressive ratio requirement assay. Shown is the mean (± SEM). Significance calculated via two-tailed Student's t test.

(D) Representative images of the nucleus accumbens (NAc) and ventral tegmental area (VTA) in VEH and ABX mice given 1 h of free access to high-sucrose pellets, with c-Fos intensity represented in green. Scale bars are 100 μm. Images are cropped to emphasize the region of interest.

(E) Density of c-Fos+ neurons in the NAc shell, NAc core, and VTA (n = 8/group) after 1 h of access to high-sucrose pellets. Shown is the mean (± SEM). Significance calculated via two-way ANOVA with microbiota status and access to high-sucrose pellets as factors, followed by Bonferroni's multiple comparisons test (within brain regions). Data for mice not given access to high-sucrose pellets is shown in Figures S3A and S3B.

See also Figures S2 and S3.

(Figures S2O and S2P). Successfully trained animals underwent progressive ratio (PR) breakpoint testing, in which ABX mice completed greater ratio requirements than VEH mice to receive a high-sucrose pellet (Figure 2C), suggesting the microbiota suppresses motivation to pursue a food reward.

Activity in reward-related brain regions is affected by the gut microbiota

The perceived incentive salience of a reward is associated with activity in the mesolimbic dopaminergic neural system.[2,3,59–61] In mice that were given 1 h of access to high-sucrose pellets, ABX treatment significantly augmented the level of brain activity observed in the ventral tegmental area (VTA), nucleus accumbens (NAc) core, and NAc shell compared with VEH controls, as a likely consequence of consuming a greater number of high-sucrose rewards (Figures 2D and 2E). VEH and ABX mice that did not receive high-sucrose pellets did not exhibit differences in neural activity in the same regions (Figures S3A and S3B). In contrast to the VTA and NAc, there was no significant effect of microbiota status on high-sucrose pellet-induced brain activity in the dorsal striatum, lateral hypothalamus (LH), or basolateral amygdala (BLA) (Figures S3C and S3D). Furthermore, there were no differences in baseline neuronal activity of homeostatic hunger-encoding neuropeptide Y-expressing (*NPY*+) neurons in the arcuate nucleus of the hypothalamus[62,63] (Figures S3E and S3F) or in expression of hypothalamic neuropeptides that vary with homeostatic need[64–66] (Figure S3G), suggesting changes in energy balance are unlikely to mediate gut microbial regulation of palatable food intake. These results demonstrate that the microbiota influences neural activity in reward-related brain regions in mice administered high-sucrose pellets, but whether these effects are required for overconsumption of palatable foods remains unknown.

Specific microbial taxa associate with suppression of high-sucrose pellet intake

The gut microbiota contains various bacterial taxa with specialized functions.[67] We sought to identify if hedonic feeding suppression is a general property of the gut microbiota or specific to certain bacterial species. Mice were administered individual antibiotics from the ABX mixture, each having a different spectrum of antimicrobial activity, and assayed for high-sucrose pellet consumption. Ampicillin (A) and vancomycin (V)-treated mice exhibited elevated consumption of high-sucrose pellets compared with VEH controls, whereas mice administered neomycin (N) or metronidazole (M) demonstrated no significant differences in intake compared with VEH mice (Figures 3A and 3B).

To verify functional changes to the gut microbiota, we performed fecal transplants from differentially treated antibiotic donor mice into ABX-treated recipients. Remarkably, microbiota transplants from A- or V-treated mice were insufficient to rescue increased pellet consumption behaviors compared with FMT from VEH donor mice (Figures 3C and 3D), demonstrating that specific antibiotics robustly and durably remodeled the gut microbiota to adopt an altered profile incapable of reducing host feeding behavior. The loss of function suggests microbial taxa sensitive to A and V suppress high-sucrose pellet consumption in mice.

16S ribosomal RNA gene sequencing confirmed that the antibiotic treatment modified microbiome community composition and V-, N-, and M-treated mice showed no significant change in fecal microbial load compared with VEH mice (Figures 3E,

CellPress
OPEN ACCESS

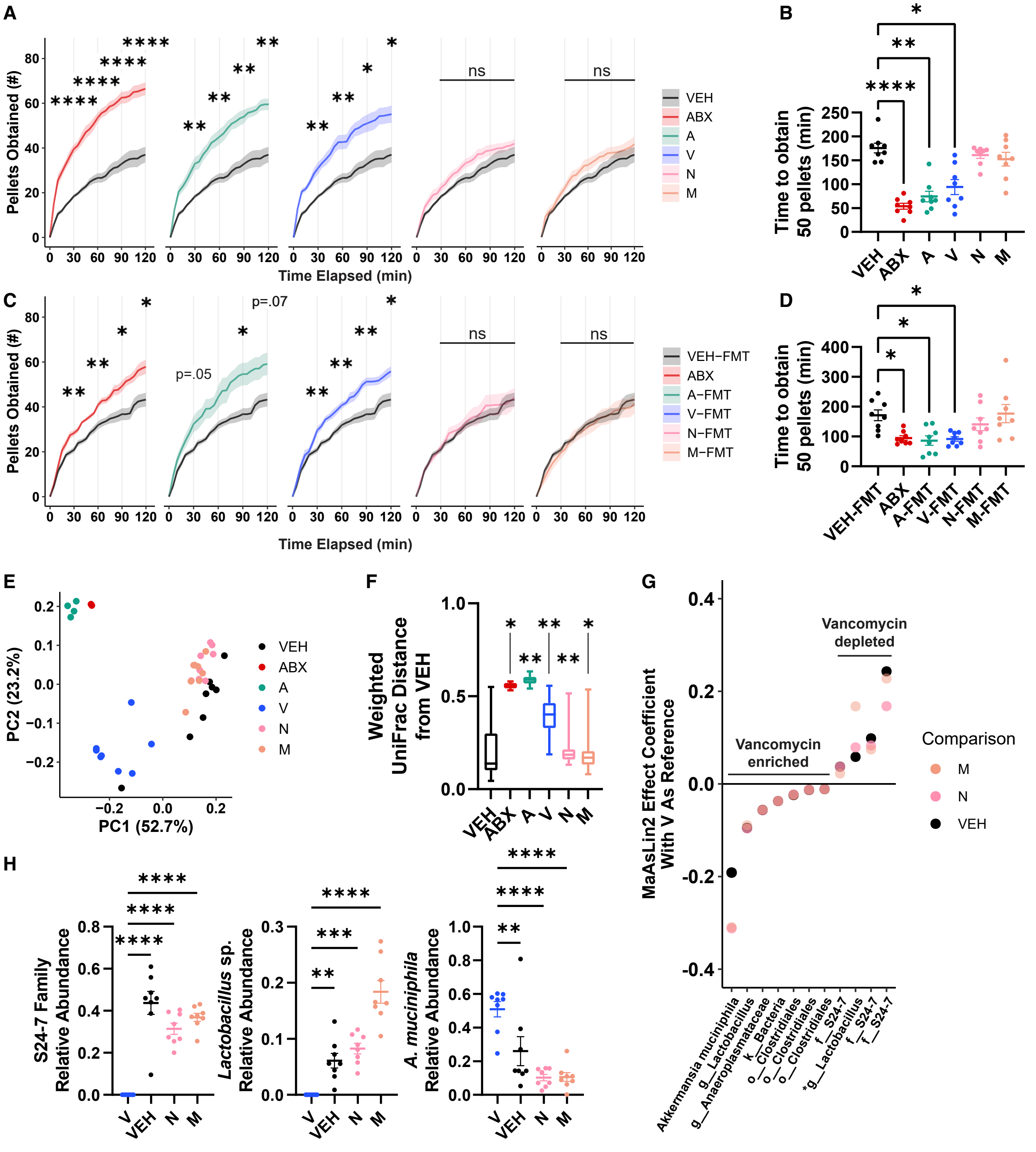

Figure 3. Certain microbial taxa correlate with suppression of high-sucrose pellet consumption

(A) Cumulative retrieval of high-sucrose pellets between mice given vehicle (VEH), combined antibiotics (ABX), or individual antibiotics (A, ampicillin; V, vancomycin; N, neomycin; M, metronidazole) (n = 8/group). Shown is the mean (± SEM) plotted every 5 min. Significance calculated via two-way repeated measures ANOVA using 30-min time points followed by Dunnett's multiple comparisons test to VEH (within time points). VEH data reproduced in each panel for reference.

(B) Times at which mice in (A) had retrieved 50 high-sucrose pellets (1 g). Significance calculated via one-way ANOVA Kruskal-Wallis followed by Dunn's multiple comparisons test to VEH.

(C) Cumulative retrieval of high-sucrose pellets between ABX-treated mice maintained on ABX or given FMT from animals administered no antibiotics or individual antibiotics (n = 8/group). Shown is the mean (± SEM) plotted every 5 min. Significance calculated via two-way repeated measures ANOVA using 30-min time points followed by Dunnett's multiple comparisons test to VEH-FMT (within time points). VEH-FMT data reproduced in each panel for reference.

(legend continued on next page)

3F, and S4A–S4D). Differential abundance analysis revealed four amplicon sequence variants (ASVs) depleted in the microbiome of V-treated mice compared with VEH, N-, and M-treated microbiomes (Figures 3G and 3H). Three of the four V-depleted ASVs aligned to members of family S24-7, a largely uncultured taxon within the order Bacteroidales,[68] and the fourth corresponded to *Lactobacillus johnsonii* and *Lactobacillus gasseri*. We isolated a strain of *Lactobacillus* from our SPF mouse colony with perfect 16S rRNA sequence identity to the differentially abundant ASV and confirmed its identity as *L. johnsonii* (Figure S4E). *Akkermansia muciniphila* was more abundant in V-treated mice compared with the intake-suppressing VEH, N, and M microbiomes, suggesting *A. muciniphila* is unlikely to suppress high-sucrose pellet intake (Figure 3H).

As the microbial features associated with reduced food intake were identified in reference to the V condition, and we aimed to capture potential microbe-microbe interactions absent in ABX mice, we used V treatment as a model to explore the effect of specific microbes on feeding behavior. Employing a limited-access binge intake assay,[19,69,70] we found that microbial transplants from SPF donor mice into V-treated mice (V + SPF) suppressed high-sucrose pellet consumption, whereas autologous FMT (V + Auto) or saline gavage (V + Sal) did not (Figures 4A and 4B). Compared with V + Auto and V + Sal mice, both V-naive (VEH) and V + SPF mice displayed distinct changes in microbiome diversity and greater relative abundances of family S24-7 and an ASV corresponding to *L. johnsonii* (Figures 4C, S4F, and S4G). To test if S24-7 and *L. johnsonii* contributed to the binge-suppressing outcome, V-treated animals were administered a fecal microbiota suspension from an SPF mouse (V + SPF), a mixture of commercially available S24-7 isolates and the previously isolated strain of *L. johnsonii* (V + 4-mix), or *A. muciniphila* as a control (V + *A. muc*) (Figure 4D). 4-mix treatment was sufficient to suppress high-sucrose pellet consumption compared with *A. muciniphila* treatment (Figure 4E). We confirmed greater abundances of S24-7 and an ASV corresponding to *L. johnsonii* in the V + 4-mix treatment group compared with V + *A. muc* mice (Figure 4F), with significant effects on microbial diversity (Figures S4H–S4I). We conclude that specific members of the commensal gut microbiota can suppress feeding behavior in mice induced by a palatable food.

DISCUSSION

Herein, we reveal that the gut microbiota reduces feeding induced in response to various palatable foods in mice. We find the gut microbiota diminishes the incentive salience of high-sucrose pellets and regulates activity in reward-related brain regions. Gut community profiling exposed microbial taxa associated with feeding suppression, and S24-7 family members and *L. johnsonii* were sufficient to reduce binge intake in an antibiotic-treatment model of overconsumption.

We found that microbiota-depleted mice overconsumed high-sucrose pellets, a HFD, and Ensure, suggesting our observations may generalize to other rewarding foods. Indeed, a recent report has demonstrated an antibiotic-induced increase in binge-like consumption of a high-fat high-sugar diet in mice.[9] Although these sweet and fat stimuli model the processed diets contributing to disease in current Western populations,[71] their composite nature limits our ability to draw conclusions about specific dietary properties required for microbiota-dependent changes in feeding. Targeted experiments involving foods with controlled levels of sweetness and fat, coupled with sensory pathway intervention, are needed to define these relationships. Pertinently, two-bottle tests reveal GF mice overconsume sucrose solutions and fat emulsions and differentially express lingual fat-detection proteins compared with SPF controls.[35,72]

We observed microbiota-dependent changes in neural activity in the VTA and NAc, regions associated with hedonic feeding,[2,3,15] in line with reports that microbiota perturbations may affect brain activity.[73–75] Central regulators of palatable food intake, including dopamine, brain-derived neurotrophic factor, and endocannabinoids, differ in GF mice compared with mice with intact microbiotas[76–78] and may regulate reward pathways that influence microbiota-mediated effects on palatable food consumption. Future research will explore specific molecular pathways linking the gut microbiota to mesolimbic brain activity.

Microbes from the S24-7 family and *L. johnsonii* are sufficient to suppress high-sucrose pellet intake in our model system, compared with treatment with *A. muciniphila*. Intriguingly, a strain of *Bacteroides uniformis*, of the same phylogenetic order as S24-7, can suppress binge eating in mice, and multiple species of *Lactobacillus* are reported to affect metabolism and feeding.[10,79–81] A next stage of this research will define the mechanisms required for gut microbes to suppress palatable food consumption.

Altered gut microbiome profiles have been associated with human eating disorders, including anorexia nervosa and binge-eating disorder,[82–86] as well as in rodent studies of palatable food intake, dietary preference, and eating disorder models.[9–11,87–89] Our findings contribute new insights to

(D) Times at which mice in (C) had retrieved 50 high-sucrose pellets (1 g). Significance calculated via one-way ANOVA Kruskal-Wallis followed by Dunn's multiple comparisons test to VEH-FMT.

(E and F) PCoA and boxplot of pairwise comparisons of weighted UniFrac distances in VEH (n = 8), ABX (n = 2), A (n = 4), V (n = 8), N (n = 8), and M (n = 8) animals. Significance calculated via permutational analaysis of variance (PERMANOVA) (Table S1). Asterisks in (F) denote significance of the PERMANOVA test of treatment groups compared with VEH.

(G) Summary of ASVs and the highest-resolution taxonomic classification of each that significantly associate with vancomycin treatment in all three comparisons against metronidazole-, neomycin-, and vehicle-treated animals, filtered for those with a >0.01 absolute MaAsLin2 effect coefficient. The ASV corresponding to *Lactobacillus* sp. is denoted with an asterisk (*). Significance calculated using a general linear model in MaAsLin2 with antibiotic as a fixed effect and cage as a random effect. False discovery rate threshold was set to 0.1. Significance values are reported in Table S2.

(H) Relative abundances of select taxa that significantly negatively (S24-7 family and *Lactobacillus* sp.) and positively (*A. muciniphila*) associate with vancomycin across all three comparisons as determined by MaAsLin2 analysis. Shown is the mean (± SEM). Significance illustrated via one-way ANOVA followed by Dunnett's multiple comparisons test to V.

See also Figure S4 and Table S2.

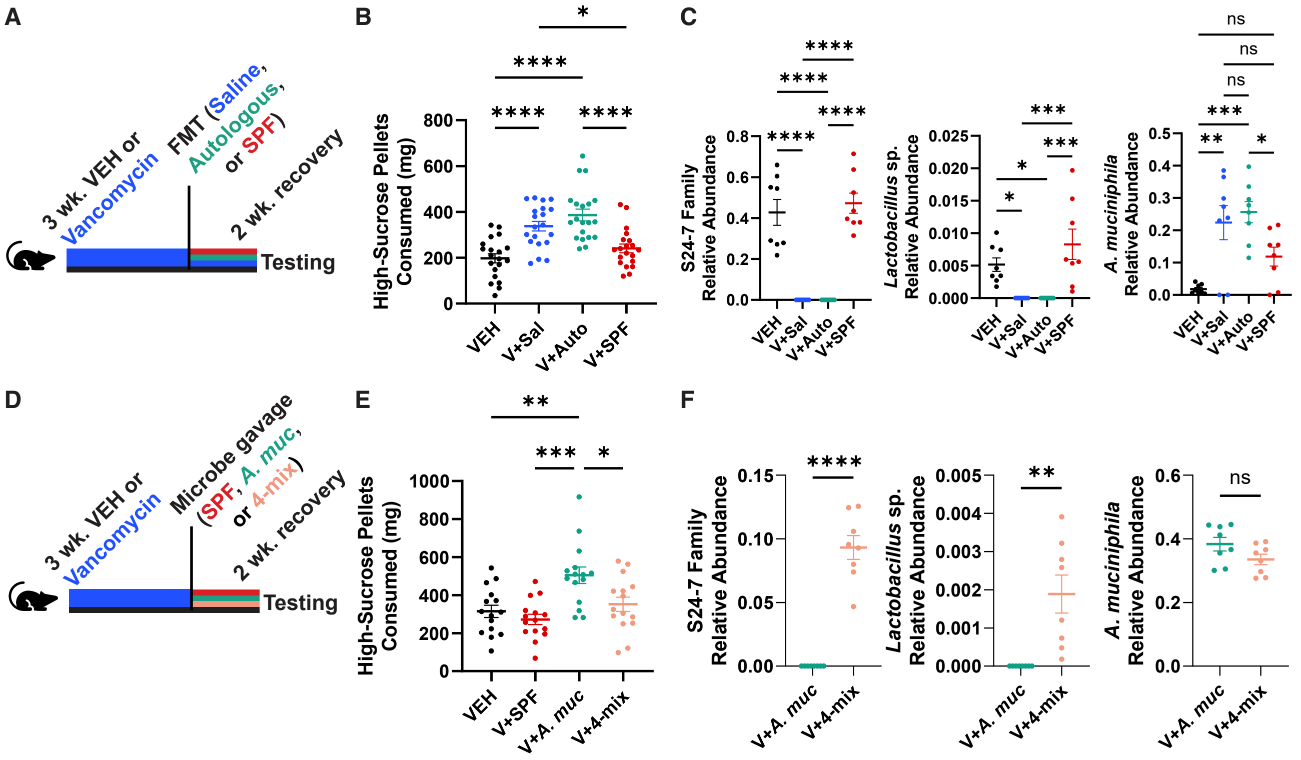

Figure 4. Family S24-7 and *L. johnsonii* functionally alter feeding in an induced model of binge-like intake

(A) Schematic illustrating timeline of vehicle (VEH) or vancomycin (V) treatment and removal, treatment with saline vehicle (V + Sal), autologous FMT (V + Auto), or FMT from an SPF donor (V + SPF), and testing of binge intake. VEH mice received saline gavages.

(B) Total 1 h intake of high-sucrose pellets in VEH, V + Sal, V + Auto, and V + SPF mice (n = 20/group). Shown is the mean (± SEM). Significance calculated via one-way ANOVA followed by Tukey's multiple comparisons test.

(C) Relative abundances of S24-7 family, *Lactobacillus* sp., and *A. muciniphila* in VEH, V + Sal, V + Auto, and V + SPF mice (n = 8/group). Shown is the mean (± SEM). Significance calculated via one-way ANOVA followed by Tukey's multiple comparisons test.

(D) Schematic illustrating timeline of vehicle (VEH) or vancomycin treatment and removal, microbial treatment with an SPF microbiota (V + SPF), *A. muciniphila* (V + *A. muc*), or a mixture of three S24-7 family members and *Lactobacillus johnsonii* (V + 4-mix), and testing of binge intake. VEH mice received saline gavages.

(E) Total 1 h intake of high-sucrose pellets in VEH, V + SPF, V + *A. muc*, and V + 4-mix mice (n = 15/group). Shown is the mean (± SEM). Significance calculated via one-way ANOVA followed by Tukey's multiple comparisons test.

(F) Relative abundances of S24-7 family, *Lactobacillus* sp., and *A. muciniphila* in V + *A. muc* and V + 4-mix mice (n = 8/group). Shown is the mean (± SEM). Significance calculated via two-tailed Student's t tests.

See also Figure S4.

growing evidence for functional gut microbiota modulation of host feeding behavior and identify candidate species for further study.[10,12,13]

STAR★METHODS

Detailed methods are provided in the online version of this paper and include the following:

- KEY RESOURCES TABLE
- RESOURCE AVAILABILITY
  - Lead contact
  - Materials availability
  - Data and code availability
- EXPERIMENTAL MODEL AND SUBJECT DETAILS
  - Mice
  - Bacterial culture conditions
- METHOD DETAILS
  - Antibiotic (ABX) treatment
  - Microbiota transplant and microbial treatment
  - Short-chain fatty acid treatment
  - Colony forming unit (CFU) analyses
  - Analysis of fecal microbial load
  - Fecal microbiome community profiling
  - Isolation of *Lactobacillus johnsonii*
  - Hypothalamic neuropeptide expression
  - Brain sample collection
  - Brain sectioning and c-Fos measurement
  - Microscopic imaging and cell quantification
  - Free-feeding intake of high-sucrose pellets
  - Free-feeding intake of a high-fat diet
  - Free-feeding intake of Ensure®
  - Bout structure analyses
  - Operant conditioning
  - Brief-access dietary selection assay
  - High-sucrose pellet binge-like consumption assay

- ○ Fasting-refeeding assays
- ○ Homeostatic food intake measurements
- ○ Baited and unbaited hole board assay
- ○ Anxiety assays
- QUANTIFICATION AND STATISTICAL ANALYSIS

SUPPLEMENTAL INFORMATION

Supplemental information can be found online at https://doi.org/10.1016/j.cub.2022.10.066.

ACKNOWLEDGMENTS

The authors thank Dr. Gil Sharon for help with animal behavioral testing and analysis of sequencing data. We are grateful to Drs. Yuki Oka, Viviana Gradinaru, and Scott Kanoski for scientific discussion. We thank Taren Thron, Yvette Garcia-Flores, Mark Adame, and Nikki Cruz for technical and logistical assistance. We thank the Caltech Office of Laboratory Animal Resources (OLAR) and the Institutional Animal Care and Use Committee (IACUC). We acknowledge the Caltech Biological Imaging Facility for microscopy assistance and the Caltech Neurotechnology Laboratory and Caltech TechLab 3D Printing Facility for behavioral equipment fabrication. We thank Dr. Catherine Oikonomou and all members of the Mazmanian Lab for critical evaluation of the manuscript. This work was supported by the National Science Foundation Graduate Research Fellowship under grant no. DGE-1745301 to J.O., the Gates Millennium Scholarship Program to J.O., and funding from the Heritage Medical Research Institute to S.K.M.

AUTHOR CONTRIBUTIONS

Conceptualization, J.O. and S.K.M.; investigation, J.O.; formal analysis, J.O. and J.C.B.; writing, J.O.; review, all authors.

DECLARATION OF INTERESTS

The authors declare no competing interests.

Received: January 19, 2022
Revised: August 30, 2022
Accepted: October 28, 2022
Published: November 29, 2022

REFERENCES

1. Coll, A.P., Farooqi, I.S., and O'Rahilly, S. (2007). The hormonal control of food intake. Cell *129*, 251–262. https://doi.org/10.1016/j.cell.2007.04.001.
2. Watts, A.G., Kanoski, S.E., Sanchez-Watts, G., and Langhans, W. (2022). The physiological control of eating: signals, neurons, and networks. Physiol. Rev. *102*, 689–813.
3. Saper, C.B., Chou, T.C., and Elmquist, J.K. (2002). The need to feed: homeostatic and hedonic control of eating. Neuron *36*, 199–211. https://doi.org/10.1016/S0896-6273(02)00969-8.
4. Cummings, D.E., and Overduin, J. (2007). Gastrointestinal regulation of food intake. J. Clin. Invest. *117*, 13–23. https://doi.org/10.1172/JCI30227.
5. Berthoud, H.R., Albaugh, V.L., and Neuhuber, W.L. (2021). Gut-brain communication and obesity: understanding functions of the vagus nerve. J. Clin. Invest. *131*, e143770. https://doi.org/10.1172/JCI143770.
6. Yu, K.B., and Hsiao, E.Y. (2021). Roles for the gut microbiota in regulating neuronal feeding circuits. J. Clin. Invest. *131*, e143772. https://doi.org/10.1172/JCI143772.
7. Bäckhed, F., Ding, H., Wang, T., Hooper, L.V., Koh, G.Y., Nagy, A., Semenkovich, C.F., and Gordon, J.I. (2004). The gut microbiota as an environmental factor that regulates fat storage. Proc. Natl. Acad. Sci. USA *101*, 15718–15723. https://doi.org/10.1073/pnas.0407076101.
8. Zarrinpar, A., Chaix, A., Xu, Z.Z., Chang, M.W., Marotz, C.A., Saghatelian, A., Knight, R., and Panda, S. (2018). Antibiotic-induced microbiome depletion alters metabolic homeostasis by affecting gut signaling and colonic metabolism. Nat. Commun. *9*, 2872. https://doi.org/10.1038/s41467-018-05336-9.
9. de Wouters d'Oplinter, A., Rastelli, M., Van Hul, M., Delzenne, N.M., Cani, P.D., and Everard, A. (2021). Gut microbes participate in food preference alterations during obesity. Gut Microbes *13*, 1959242. https://doi.org/10.1080/19490976.2021.1959242.
10. Agustí, A., Campillo, I., Balzano, T., Benítez-Páez, A., López-Almela, I., Romaní-Pérez, M., Forteza, J., Felipo, V., Avena, N.M., and Sanz, Y. (2021). Bacteroides uniformis CECT 7771 modulates the brain reward response to reduce binge eating and anxiety-like behavior in rat. Mol. Neurobiol. *58*, 4959–4979. https://doi.org/10.1007/s12035-021-02462-2.
11. Trevelline, B.K., and Kohl, K.D. (2022). The gut microbiome influences host diet selection behavior. Proc. Natl. Acad. Sci. USA *119*. e2117537119. https://doi.org/10.1073/pnas.2117537119.
12. Gabanyi, I., Lepousez, G., Wheeler, R., Vieites-Prado, A., Nissant, A., Wagner, S., Moigneu, C., Dulauroy, S., Hicham, S., Polomack, B., et al. (2022). Bacterial sensing via neuronal Nod2 regulates appetite and body temperature. Science *376*, eabj3986. https://doi.org/10.1126/science.abj3986.
13. Petrovich, G.D. (2018). Feeding behavior survival circuit: anticipation & competition. Curr. Opin. Behav. Sci. *24*, 137–142. https://doi.org/10.1016/j.cobeha.2018.09.007.
14. Johnson, A.W. (2013). Eating beyond metabolic need: how environmental cues influence feeding behavior. Trends Neurosci. *36*, 101–109. https://doi.org/10.1016/j.tins.2013.01.002.
15. Liu, C.M., and Kanoski, S.E. (2018). Homeostatic and non-homeostatic controls of feeding behavior: distinct vs. common neural systems. Physiol. Behav. *193*, 223–231. https://doi.org/10.1016/j.physbeh.2018.02.011.
16. Rossi, M.A., and Stuber, G.D. (2018). Overlapping brain circuits for homeostatic and hedonic feeding. Cell Metab. *27*, 42–56. https://doi.org/10.1016/j.cmet.2017.09.021.
17. Berridge, K.C. (1996). Food reward: brain substrates of wanting and liking. Neurosci. Biobehav. Rev. *20*, 1–25. https://doi.org/10.1016/0149-7634(95)00033-B.
18. Yasoshima, Y., and Shimura, T. (2015). A mouse model for binge-like sucrose overconsumption: contribution of enhanced motivation for sweetener consumption. Physiol. Behav. *138*, 154–164. https://doi.org/10.1016/j.physbeh.2014.10.035.
19. Babbs, R.K., Kelliher, J.C., Scotellaro, J.L., Luttik, K.P., Mulligan, M.K., and Bryant, C.D. (2018). Genetic differences in the behavioral organization of binge eating, conditioned food reward, and compulsive-like eating in C57BL/6J and DBA/2J strains. Physiol. Behav. *197*, 51–66. https://doi.org/10.1016/j.physbeh.2018.09.013.
20. Corwin, R.L.W., and Babbs, R.K. (2012). Rodent models of binge eating: are they models of addiction? ILAR J. *53*, 23–34. https://doi.org/10.1093/ilar.53.1.23.
21. Davis, J.D. (1989). The microstructure of ingestive behavior. Ann. NY Acad. Sci. *575*, 106–119. , discussion 120. https://doi.org/10.1111/j.1749-6632.1989.tb53236.x.
22. Spector, A.C., and St John, S.J. (1998). Role of taste in the microstructure of quinine ingestion by rats. Am. J. Physiol. *274*, R1687–R1703. https://doi.org/10.1152/ajpregu.1998.274.6.R1687.
23. Yeomans, M.R. (1998). Taste, palatability and the control of appetite. Proc. Nutr. Soc. *57*, 609–615. https://doi.org/10.1079/PNS19980089.
24. Davis, J.D., and Perez, M.C. (1993). Food deprivation- and palatability-induced microstructural changes in ingestive behavior. Am. J. Physiol. *264*, R97–R103. https://doi.org/10.1152/ajpregu.1993.264.1.R97.
25. Davis, J.D., and Levine, M.W. (1977). A model for the control of ingestion. Psychol. Rev. *84*, 379–412. https://doi.org/10.1037/0033-295X.84.4.379.
26. Sclafani, A., and Ackroff, K. (2003). Reinforcement value of sucrose measured by progressive ratio operant licking in the rat. Physiol. Behav. *79*, 663–670. https://doi.org/10.1016/S0031-9384(03)00143-4.

27. Guttman, N. (1953). Operant conditioning, extinction, and periodic reinforcement in relation to concentration of sucrose used as reinforcing agent. J. Exp. Psychol. *46*, 213–224. https://doi.org/10.1037/h0061893.

28. Sharma, S., Hryhorczuk, C., and Fulton, S. (2012). Progressive-ratio responding for palatable high-fat and high-sugar food in mice. J. Vis. Exp. e3754.

29. Denis, R.P., Joly-Amado, A., Webber, E., Langlet, F., Schaeffer, M., Padilla, S., Cansell, C., Dehouck, B., Castel, J., Delbés, A.-S., et al. (2015). Palatability can drive feeding independent of AgRP neurons. Cell Metab. *22*, 646–657. https://doi.org/10.1016/j.cmet.2015.07.011.

30. Li, Z., Yi, C.X., Katiraei, S., Kooijman, S., Zhou, E., Chung, C.K., Gao, Y., van den Heuvel, J.K., Meijer, O.C., Berbée, J.F.P., et al. (2018). Butyrate reduces appetite and activates brown adipose tissue via the gut-brain neural circuit. Gut *67*, 1269–1279. https://doi.org/10.1136/gutjnl-2017-314050.

31. Frost, G., Sleeth, M.L., Sahuri-Arisoylu, M., Lizarbe, B., Cerdan, S., Brody, L., Anastasovska, J., Ghourab, S., Hankir, M., Zhang, S., et al. (2014). The short-chain fatty acid acetate reduces appetite via a central homeostatic mechanism. Nat. Commun. *5*, 3611. https://doi.org/10.1038/ncomms4611.

32. Niimi, K., and Takahashi, E. (2019). New system to examine the activity and water and food intake of germ-free mice in a sealed positive-pressure cage. Heliyon *5*. e02176. https://doi.org/10.1016/j.heliyon.2019.e02176.

33. Moretti, C.H., Schiffer, T.A., Li, X., Weitzberg, E., Carlström, M., and Lundberg, J.O. (2021). Germ-free mice are not protected against diet-induced obesity and metabolic dysfunction. Acta Physiol. (Oxf) *231*. e13581. https://doi.org/10.1111/apha.13581.

34. Rabot, S., Membrez, M., Bruneau, A., Gérard, P., Harach, T., Moser, M., Raymond, F., Mansourian, R., and Chou, C.J. (2010). Germ-free C57BL/6J mice are resistant to high-fat-diet-induced insulin resistance and have altered cholesterol metabolism. FASEB J. *24*, 4948–4959. https://doi.org/10.1096/fj.10-164921.

35. Swartz, T.D., Duca, F.A., Wouters, T. de, Sakar, Y., and Covasa, M. (2012). Up-regulation of intestinal type 1 taste receptor 3 and sodium glucose luminal transporter-1 expression and increased sucrose intake in mice lacking gut microbiota. Br. J. Nutr. *107*, 621–630. https://doi.org/10.1017/S0007114511003412.

36. Muller, P.A., Schneeberger, M., Matheis, F., Wang, P., Kerner, Z., Ilanges, A., Pellegrino, K., del Mármol, J., Castro, T.B.R., Furuichi, M., et al. (2020). Microbiota modulate sympathetic neurons via a gut–brain circuit. Nature *583*, 441–446. https://doi.org/10.1038/s41586-020-2474-7.

37. Schieber, A.M.P., Lee, Y.M., Chang, M.W., Leblanc, M., Collins, B., Downes, M., Evans, R.M., and Ayres, J.S. (2015). Disease tolerance mediated by microbiome *E. coli* involves inflammasome and IGF-1 signaling. Science *350*, 558–563. https://doi.org/10.1126/science.aac6468.

38. Wu, W.L., Adame, M.D., Liou, C.W., Barlow, J.T., Lai, T.T., Sharon, G., Schretter, C.E., Needham, B.D., Wang, M.I., Tang, W., et al. (2021). Microbiota regulate social behaviour via stress response neurons in the brain. Nature *595*, 409–414. https://doi.org/10.1038/s41586-021-03669-y.

39. Desbonnet, L., Clarke, G., Traplin, A., O'Sullivan, O., Crispie, F., Moloney, R.D., Cotter, P.D., Dinan, T.G., and Cryan, J.F. (2015). Gut microbiota depletion from early adolescence in mice: implications for brain and behaviour. Brain Behav. Immun. *48*, 165–173. https://doi.org/10.1016/j.bbi.2015.04.004.

40. Wu, J.T., Sun, C.L., Lai, T.T., Liou, C.W., Lin, Y.Y., Xue, J.Y., Wang, H.W., Chai, L.M.X., Lee, Y.J., Chen, S.L., et al. (2022). Oral short-chain fatty acids administration regulates innate anxiety in adult microbiome-depleted mice. Neuropharmacology *214*, 109140. https://doi.org/10.1016/j.neuropharm.2022.109140.

41. Chu, C., Murdock, M.H., Jing, D., Won, T.H., Chung, H., Kressel, A.M., Tsaava, T., Addorisio, M.E., Putzel, G.G., Zhou, L., et al. (2019). The microbiota regulate neuronal function and fear extinction learning. Nature *574*, 543–548. https://doi.org/10.1038/s41586-019-1644-y.

42. Nguyen, K.P., O'Neal, T.J., Bolonduro, O.A., White, E., and Kravitz, A.V. (2016). Feeding experimentation device (FED): a flexible open-source device for measuring feeding behavior. J. Neurosci. Methods *267*, 108–114. https://doi.org/10.1016/j.jneumeth.2016.04.003.

43. Goulding, E.H., Schenk, A.K., Juneja, P., MacKay, A.W., Wade, J.M., and Tecott, L.H. (2008). A robust automated system elucidates mouse home cage behavioral structure. Proc. Natl. Acad. Sci. USA *105*, 20575–20582. https://doi.org/10.1073/pnas.0809053106.

44. Rathod, Y.D., and Di Fulvio, M.D. (2021). The feeding microstructure of male and female mice. PLoS One *16*, e0246569. https://doi.org/10.1371/journal.pone.0246569.

45. Crawley, J.N. (1985). Exploratory behavior models of anxiety in mice. Neurosci. Biobehav. Rev. *9*, 37–44. https://doi.org/10.1016/0149-7634(85)90030-2.

46. Burnett, C.J., Li, C., Webber, E., Tsaousidou, E., Xue, S.Y., Brüning, J.C., and Krashes, M.J. (2016). Hunger-driven motivational state competition. Neuron *92*, 187–201. https://doi.org/10.1016/j.neuron.2016.08.032.

47. Samuels, B.A., and Hen, R. (2011). Novelty-suppressed feeding in the mouse. In Mood and Anxiety Related Phenotypes in Mice Neuromethods, T.D. Gould, ed. (Humana Press), pp. 107–121. https://doi.org/10.1007/978-1-61779-313-4_7.

48. Walf, A.A., and Frye, C.A. (2007). The use of the elevated plus maze as an assay of anxiety-related behavior in rodents. Nat. Protoc. *2*, 322–328. https://doi.org/10.1038/nprot.2007.44.

49. Gould, T.D., Dao, D.T., and Kovacsics, C.E. (2009). The open field test. In Mood and Anxiety Related Phenotypes in Mice: Characterization Using Behavioral Tests Neuromethods, T.D. Gould, ed. (Humana Press), pp. 1–20.

50. Hoban, A.E., Moloney, R.D., Golubeva, A.V., McVey Neufeld, K.A., O'Sullivan, O., Patterson, E., Stanton, C., Dinan, T.G., Clarke, G., and Cryan, J.F. (2016). Behavioural and neurochemical consequences of chronic gut microbiota depletion during adulthood in the rat. Neuroscience *339*, 463–477. https://doi.org/10.1016/j.neuroscience.2016.10.003.

51. Fröhlich, E.E., Farzi, A., Mayerhofer, R., Reichmann, F., Jačan, A., Wagner, B., Zinser, E., Bordag, N., Magnes, C., Fröhlich, E., et al. (2016). Cognitive impairment by antibiotic-induced gut dysbiosis: analysis of gut microbiota-brain communication. Brain Behav. Immun. *56*, 140–155. https://doi.org/10.1016/j.bbi.2016.02.020.

52. McDougle, M., Quinn, D., Diepenbroek, C., Singh, A., de la Serre, C., and de Lartigue, G. (2021). Intact vagal gut-brain signalling prevents hyperphagia and excessive weight gain in response to high-fat high-sugar diet. Acta Physiol. (Oxf) *231*. e13530. https://doi.org/10.1111/apha.13530.

53. Chambers, A.P., Sandoval, D.A., and Seeley, R.J. (2013). Integration of satiety signals by the central nervous system. Curr. Biol. *23*, R379–R388. https://doi.org/10.1016/j.cub.2013.03.020.

54. Siemian, J.N., Arenivar, M.A., Sarsfield, S., Borja, C.B., Russell, C.N., and Aponte, Y. (2021). Lateral hypothalamic LEPR neurons drive appetitive but not consummatory behaviors. Cell Rep. *36*, 109615. https://doi.org/10.1016/j.celrep.2021.109615.

55. Jones, S.R., and Fordahl, S.C. (2021). Bingeing on high-fat food enhances evoked dopamine release and reduces dopamine uptake in the nucleus accumbens. Obesity (Silver Spring) *29*, 721–730. https://doi.org/10.1002/oby.23122.

56. Campos, C.A., Bowen, A.J., Schwartz, M.W., and Palmiter, R.D. (2016). Parabrachial CGRP neurons control meal termination. Cell Metab. *23*, 811–820. https://doi.org/10.1016/j.cmet.2016.04.006.

57. Le Roy, T., Debédat, J., Marquet, F., Da-Cunha, C., Ichou, F., Guerre-Millo, M., Kapel, N., Aron-Wisnewsky, J., and Clément, K. (2018). Comparative evaluation of microbiota engraftment following fecal microbiota transfer in mice models: age, kinetic and microbial status matter. Front. Microbiol. *9*, 3289. https://doi.org/10.3389/fmicb.2018.03289.

58. Matikainen-Ankney, B.A., Earnest, T., Ali, M., Casey, E., Wang, J.G., Sutton, A.K., Legaria, A.A., Barclay, K.M., Murdaugh, L.B., Norris, M.R., et al. (2021). An open-source device for measuring food intake

and operant behavior in rodent home-cages. eLife *10*, e66173. https://doi.org/10.7554/eLife.66173.

59. Kutlu, M.G., Zachry, J.E., Melugin, P.R., Cajigas, S.A., Chevee, M.F., Kelly, S.J., Kutlu, B., Tian, L., Siciliano, C.A., and Calipari, E.S. (2021). Dopamine release in the nucleus accumbens core signals perceived saliency. Curr. Biol. *31*. 4748.e8–4761.e8. https://doi.org/10.1016/j.cub.2021.08.052.

60. Hsu, T.M., McCutcheon, J.E., and Roitman, M.F. (2018). Parallels and overlap: the integration of homeostatic signals by mesolimbic dopamine neurons. Front. Psychiatry *9*, 410. https://doi.org/10.3389/fpsyt.2018.00410.

61. Berridge, K.C. (2007). The debate over dopamine's role in reward: the case for incentive salience. Psychopharmacology (Berl.) *191*, 391–431. https://doi.org/10.1007/s00213-006-0578-x.

62. Hahn, T.M., Breininger, J.F., Baskin, D.G., and Schwartz, M.W. (1998). Coexpression of Agrp and NPY in fasting-activated hypothalamic neurons. Nat. Neurosci. *1*, 271–272.

63. Augustine, V., Lee, S., and Oka, Y. (2020). Neural control and modulation of thirst, sodium appetite, and hunger. Cell *180*, 25–32. https://doi.org/10.1016/j.cell.2019.11.040.

64. Mizuno, T.M., and Mobbs, C.V. (1999). Hypothalamic agouti-related protein messenger ribonucleic acid is inhibited by leptin and stimulated by fasting. Endocrinology *140*, 814–817. https://doi.org/10.1210/endo.140.2.6491.

65. Sanacora, G., Kershaw, M., Finkelstein, J.A., and White, J.D. (1990). Increased hypothalamic content of preproneuropeptide Y messenger ribonucleic acid in genetically obese Zucker rats and its regulation by food deprivation. Endocrinology *127*, 730–737. https://doi.org/10.1210/endo-127-2-730.

66. Perello, M., Stuart, R.C., and Nillni, E.A. (2007). Differential effects of fasting and leptin on proopiomelanocortin peptides in the arcuate nucleus and in the nucleus of the solitary tract. Am. J. Physiol. Endocrinol. Metab. *292*, E1348–E1357. https://doi.org/10.1152/ajpendo.00466.2006.

67. Donaldson, G.P., Lee, S.M., and Mazmanian, S.K. (2016). Gut biogeography of the bacterial microbiota. Nat. Rev. Microbiol. *14*, 20–32. https://doi.org/10.1038/nrmicro3552.

68. Ormerod, K.L., Wood, D.L.A., Lachner, N., Gellatly, S.L., Daly, J.N., Parsons, J.D., Dal'Molin, C.G.O., Palfreyman, R.W., Nielsen, L.K., Cooper, M.A., et al. (2016). Genomic characterization of the uncultured Bacteroidales family S24-7 inhabiting the guts of homeothermic animals. Microbiome *4*, 36. https://doi.org/10.1186/s40168-016-0181-2.

69. Kirkpatrick, S.L., Goldberg, L.R., Yazdani, N., Babbs, R.K., Wu, J., Reed, E.R., Jenkins, D.F., Bolgioni, A.F., Landaverde, K.I., Luttik, K.P., et al. (2017). Cytoplasmic FMR1-interacting protein 2 is a major genetic factor underlying binge eating. Biol. Psychiatry *81*, 757–769. https://doi.org/10.1016/j.biopsych.2016.10.021.

70. Castro, D.C., Oswell, C.S., Zhang, E.T., Pedersen, C.E., Piantadosi, S.C., Rossi, M.A., Hunker, A.C., Guglin, A., Morón, J.A., Zweifel, L.S., et al. (2021). An endogenous opioid circuit determines state-dependent reward consumption. Nature *598*, 646–651.

71. Cordain, L., Eaton, S.B., Sebastian, A., Mann, N., Lindeberg, S., Watkins, B.A., O'Keefe, J.H., and Brand-Miller, J. (2005). Origins and evolution of the Western diet: health implications for the 21st century. Am. J. Clin. Nutr. *81*, 341–354. https://doi.org/10.1093/ajcn.81.2.341.

72. Duca, F.A., Swartz, T.D., Sakar, Y., and Covasa, M. (2012). Increased oral detection, but decreased intestinal signaling for fats in mice lacking gut microbiota. PLoS One *7*, e39748. https://doi.org/10.1371/journal.pone.0039748.

73. García-Cabrerizo, R., Carbia, C., O Riordan, K.J., Schellekens, H., and Cryan, J.F. (2021). Microbiota-gut-brain axis as a regulator of reward processes. J. Neurochem. *157*, 1495–1524. https://doi.org/10.1111/jnc.15284.

74. Crumeyrolle-Arias, M., Jaglin, M., Bruneau, A., Vancassel, S., Cardona, A., Daugé, V., Naudon, L., and Rabot, S. (2014). Absence of the gut microbiota enhances anxiety-like behavior and neuroendocrine response to acute stress in rats. Psychoneuroendocrinology *42*, 207–217. https://doi.org/10.1016/j.psyneuen.2014.01.014.

75. Diaz Heijtz, R.D., Wang, S., Anuar, F., Qian, Y., Björkholm, B., Samuelsson, A., Hibberd, M.L., Forssberg, H., and Pettersson, S. (2011). Normal gut microbiota modulates brain development and behavior. Proc. Natl. Acad. Sci. USA *108*, 3047–3052. https://doi.org/10.1073/pnas.1010529108.

76. Nishino, R., Mikami, K., Takahashi, H., Tomonaga, S., Furuse, M., Hiramoto, T., Aiba, Y., Koga, Y., and Sudo, N. (2013). Commensal microbiota modulate murine behaviors in a strictly contamination-free environment confirmed by culture-based methods. Neurogastroenterol. Motil. *25*, 521–528. https://doi.org/10.1111/nmo.12110.

77. Manca, C., Shen, M., Boubertakh, B., Martin, C., Flamand, N., Silvestri, C., and Di Marzo, V. (2020). Alterations of brain endocannabinoidome signaling in germ-free mice. Biochim. Biophys. Acta Mol. Cell Biol. Lipids *1865*, 158786. https://doi.org/10.1016/j.bbalip.2020.158786.

78. Bercik, P., Denou, E., Collins, J., Jackson, W., Lu, J., Jury, J., Deng, Y., Blennerhassett, P., Macri, J., McCoy, K.D., et al. (2011). The intestinal microbiota affect central levels of brain-derived neurotropic factor and behavior in mice. Gastroenterology *141*. 599.e1–3–609.e1–3. https://doi.org/10.1053/j.gastro.2011.04.052.

79. Valladares, R., Sankar, D., Li, N., Williams, E., Lai, K.-K., Abdelgeliel, A.S., Gonzalez, C.F., Wasserfall, C.H., iii, Larkin, J., Schatz, D., et al. (2010). Lactobacillus johnsonii N6.2 mitigates the development of type 1 diabetes in BB-DP rats. PLOS One *5*. e10507. https://doi.org/10.1371/journal.pone.0010507.

80. Kang, J.-H., Yun, S.-I., Park, M.-H., Park, J.-H., Jeong, S.-Y., and Park, H.-O. (2013). Anti-obesity effect of *Lactobacillus gasseri* BNR17 in high-sucrose diet-induced obese mice. PLoS One *8*, e54617. https://doi.org/10.1371/journal.pone.0054617.

81. Chagwedera, D.N., Ang, Q.Y., Bisanz, J.E., Leong, Y.A., Ganeshan, K., Cai, J., Patterson, A.D., Turnbaugh, P.J., and Chawla, A. (2019). Nutrient sensing in CD11c cells alters the gut microbiota to regulate food intake and body mass. Cell Metab. *30*. 364.e7–373.e7. https://doi.org/10.1016/j.cmet.2019.05.002.

82. Armougom, F., Henry, M., Vialettes, B., Raccah, D., and Raoult, D. (2009). Monitoring bacterial community of human gut microbiota reveals an increase in Lactobacillus in obese patients and methanogens in anorexic patients. PLoS One *4*, e7125. https://doi.org/10.1371/journal.pone.0007125.

83. Morita, C., Tsuji, H., Hata, T., Gondo, M., Takakura, S., Kawai, K., Yoshihara, K., Ogata, K., Nomoto, K., Miyazaki, K., and Sudo, N. (2015). Gut dysbiosis in patients with anorexia nervosa. PLoS One *10*, e0145274. https://doi.org/10.1371/journal.pone.0145274.

84. Mack, I., Cuntz, U., Grämer, C., Niedermaier, S., Pohl, C., Schwiertz, A., Zimmermann, K., Zipfel, S., Enck, P., and Penders, J. (2016). Weight gain in anorexia nervosa does not ameliorate the faecal microbiota, branched chain fatty acid profiles, and gastrointestinal complaints. Sci. Rep. *6*, 26752. https://doi.org/10.1038/srep26752.

85. Prochazkova, P., Roubalova, R., Dvorak, J., Kreisinger, J., Hill, M., Tlaskalova-Hogenova, H., Tomasova, P., Pelantova, H., Cermakova, M., Kuzma, M., et al. (2021). The intestinal microbiota and metabolites in patients with anorexia nervosa. Gut Microbes *13*, 1–25. https://doi.org/10.1080/19490976.2021.1902771.

86. Leyrolle, Q., Cserjesi, R., Mulders, M.D.G.H., Zamariola, G., Hiel, S., Gianfrancesco, M.A., Rodriguez, J., Portheault, D., Amadieu, C., Leclercq, S., et al. (2021). Specific gut microbial, biological, and psychiatric profiling related to binge eating disorders: a cross-sectional study in obese patients. Clin. Nutr. *40*, 2035–2044. https://doi.org/10.1016/j.clnu.2020.09.025.

87. Breton, J., Tirelle, P., Hasanat, S., Pernot, A., L'Huillier, C., do Rego, J.-C., Déchelotte, P., Coëffier, M., Bindels, L.B., and Ribet, D. (2021). Gut microbiota alteration in a mouse model of Anorexia Nervosa. Clin. Nutr. *40*, 181–189. https://doi.org/10.1016/j.clnu.2020.05.002.

88. Bernard, A., Ancel, D., Neyrinck, A.M., Dastugue, A., Bindels, L.B., Delzenne, N.M., and Besnard, P. (2019). A preventive prebiotic supplementation improves the sweet taste perception in diet-induced obese mice. Nutrients *11*, 549. https://doi.org/10.3390/nu11030549.

89. Lyte, M., Fodor, A.A., Chapman, C.D., Martin, G.G., Perez-Chanona, E., Jobin, C., and Dess, N.K. (2016). Gut microbiota and a selectively bred taste phenotype: a novel model of microbiome-behavior relationships. Psychosom. Med. *78*, 610–619. https://doi.org/10.1097/PSY.0000000000000318.

90. Caporaso, J.G., Lauber, C.L., Walters, W.A., Berg-Lyons, D., Lozupone, C.A., Turnbaugh, P.J., Fierer, N., and Knight, R. (2011). Global patterns of 16S rRNA diversity at a depth of millions of sequences per sample. Proc. Natl. Acad. Sci. USA *108* (*Suppl 1*), 4516–4522. https://doi.org/10.1073/pnas.1000080107.

91. Lane, D.J. (1991). 16S/23S rRNA sequencing. In Nucleic Acid Techniques in Bacterial Systematics, E. Stackebrandt, and M. Goodfellow, eds. (John Wiley and Sons), pp. 115–175.

92. Turner, S., Pryer, K.M., Miao, V.P., and Palmer, J.D. (1999). Investigating deep phylogenetic relationships among cyanobacteria and plastids by small subunit rRNA sequence analysis. J. Eukaryot. Microbiol. *46*, 327–338. https://doi.org/10.1111/j.1550-7408.1999.tb04612.x.

93. Piper, M.L., Unger, E.K., Myers, M.G., and Xu, A.W. (2008). Specific physiological roles for signal transducer and activator of transcription 3 in leptin receptor-expressing neurons. Mol. Endocrinol. *22*, 751–759. https://doi.org/10.1210/me.2007-0389.

94. Reichenbach, A., Mequinion, M., Bayliss, J.A., Lockie, S.H., Lemus, M.B., Mynatt, R.L., Stark, R., and Andrews, Z.B. (2018). Carnitine acetyltransferase in AgRP neurons is required for the homeostatic adaptation to restricted feeding in male mice. Endocrinology *159*, 2473–2483. https://doi.org/10.1210/en.2018-00131.

95. R Core Team (2018). R: a language and environment for statistical computing. (R Foundation for Statistical Computing). https://www.R-project.org/.

96. RStudio Team (2020). RStudio: integrated development for R. http://www.rstudio.com/.

97. Bolyen, E., Rideout, J.R., Dillon, M.R., Bokulich, N.A., Abnet, C.C., Al-Ghalith, G.A., Alexander, H., Alm, E.J., Arumugam, M., Asnicar, F., et al. (2019). Reproducible, interactive, scalable and extensible microbiome data science using QIIME 2. Nat. Biotechnol. *37*, 852–857. https://doi.org/10.1038/s41587-019-0209-9.

98. Noldus, L.P.J.J., Spink, A.J., and Tegelenbosch, R.A.J. (2001). EthoVision: a versatile video tracking system for automation of behavioral experiments. Behav. Res. Methods Instrum. Comput. *33*, 398–414. https://doi.org/10.3758/BF03195394.

99. Schindelin, J., Arganda-Carreras, I., Frise, E., Kaynig, V., Longair, M., Pietzsch, T., Preibisch, S., Rueden, C., Saalfeld, S., Schmid, B., et al. (2012). Fiji: an open-source platform for biological-image analysis. Nat. Methods *9*, 676–682. https://doi.org/10.1038/nmeth.2019.

100. Mallick, H., Rahnavard, A., McIver, L.J., Ma, S., Zhang, Y., Nguyen, L.H., Tickle, T.L., Weingart, G., Ren, B., Schwager, E.H., et al. (2021). Multivariable association discovery in population-scale meta-omics studies. PLoS Comput. Biol. *17*, e1009442. https://doi.org/10.1371/journal.pcbi.1009442.

101. Altschul, S.F., Gish, W., Miller, W., Myers, E.W., and Lipman, D.J. (1990). Basic local alignment search tool. J. Mol. Biol. *215*, 403–410. https://doi.org/10.1016/S0022-2836(05)80360-2.

102. Friard, O., and Gamba, M. (2016). BORIS: a free, versatile open-source event-logging software for video/audio coding and live observations. Methods Ecol. Evol. *7*, 1325–1330. https://doi.org/10.1111/2041-210X.12584.

103. Kim, S., Covington, A., and Pamer, E.G. (2017). The intestinal microbiota: antibiotics, colonization resistance, and enteric pathogens. Immunol. Rev. *279*, 90–105. https://doi.org/10.1111/imr.12563.

104. Smith, P.M., Howitt, M.R., Panikov, N., Michaud, M., Gallini, C.A., Bohlooly-Y, M., Glickman, J.N., and Garrett, W.S. (2013). The microbial metabolites, short-chain fatty acids, regulate colonic Treg cell homeostasis. Science *341*, 569–573. https://doi.org/10.1126/science.1241165.

105. Erny, D., Hrabě de Angelis, A.L., Jaitin, D., Wieghofer, P., Staszewski, O., David, E., Keren-Shaul, H., Mahlakoiv, T., Jakobshagen, K., Buch, T., et al. (2015). Host microbiota constantly control maturation and function of microglia in the CNS. Nat. Neurosci. *18*, 965–977. https://doi.org/10.1038/nn.4030.

106. Kozich, J.J., Westcott, S.L., Baxter, N.T., Highlander, S.K., and Schloss, P.D. (2013). Development of a dual-index sequencing strategy and curation pipeline for analyzing amplicon sequence data on the MiSeq Illumina sequencing platform. Appl. Environ. Microbiol. *79*, 5112–5120. https://doi.org/10.1128/AEM.01043-13.

107. Thompson, L.R., Sanders, J.G., McDonald, D., Amir, A., Ladau, J., Locey, K.J., Prill, R.J., Tripathi, A., Gibbons, S.M., Ackermann, G., et al. (2017). A communal catalogue reveals Earth's multiscale microbial diversity. Nature *551*, 457–463. https://doi.org/10.1038/nature24621.

108. Madeira, F., Park, Y.M., Lee, J., Buso, N., Gur, T., Madhusoodanan, N., Basutkar, P., Tivey, A.R.N., Potter, S.C., Finn, R.D., and Lopez, R. (2019). The EMBL-EBI search and sequence analysis tools APIs in 2019. Nucleic Acids Res. *47*, W636–W641. https://doi.org/10.1093/nar/gkz268.

109. Paxinos, G., and Franklin, K.B.J. (2019). Paxinos and Franklin's the Mouse Brain in Stereotaxic Coordinates (Academic Press).

110. Rouibi, K., and Contarino, A. (2012). Increased motivation to eat in opiate-withdrawn mice. Psychopharmacology (Berl.) *221*, 675–684. https://doi.org/10.1007/s00213-011-2612-x.

111. Blasio, A., Steardo, L., Sabino, V., and Cottone, P. (2014). Opioid system in the medial prefrontal cortex mediates binge-like eating. Addict. Biol. *19*, 652–662. https://doi.org/10.1111/adb.12033.

112. Babbs, R.K., Beierle, J.A., Yao, E.J., Kelliher, J.C., Medeiros, A.R., Anandakumar, J., Shah, A.A., Chen, M.M., Johnson, W.E., and Bryant, C.D. (2020). The effect of the demyelinating agent cuprizone on binge-like eating of sweetened palatable food in female and male C57BL/6 substrains. Appetite *150*, 104678. https://doi.org/10.1016/j.appet.2020.104678.

113. Thiele, T.E., Crabbe, J.C., and Boehm, S.L. (2014). "Drinking in the dark" (DID): a simple mouse model of binge-like alcohol intake. Curr. Protoc. Neurosci. *68*, 9.49.1–9.49.12. https://doi.org/10.1002/0471142301.ns0949s68.

114. Strohmayer, A.J., and Smith, G.P. (1987). The meal pattern of genetically obese (ob/ob) mice. Appetite *8*, 111–123. https://doi.org/10.1016/S0195-6663(87)80004-1.

115. Ford, M.M., Steele, A.M., McCracken, A.D., Finn, D.A., and Grant, K.A. (2013). The relationship between adjunctive drinking, blood ethanol concentration and plasma corticosterone across fixed-time intervals of food delivery in two inbred mouse strains. Psychoneuroendocrinology *38*, 2598–2610. https://doi.org/10.1016/j.psyneuen.2013.06.011.

116. Richardson, N.R., and Roberts, D.C.S. (1996). Progressive ratio schedules in drug self-administration studies in rats: a method to evaluate reinforcing efficacy. J. Neurosci. Methods *66*, 1–11. https://doi.org/10.1016/0165-0270(95)00153-0.

117. Devarakonda, K., Nguyen, K.P., and Kravitz, A.V. (2016). ROBucket: a low cost operant chamber based on the Arduino microcontroller. Behav. Res. Methods *48*, 503–509. https://doi.org/10.3758/s13428-015-0603-2.

118. Goldberg, L.R., Kirkpatrick, S.L., Yazdani, N., Luttik, K.P., Lacki, O.A., Babbs, R.K., Jenkins, D.F., Johnson, W.E., and Bryant, C.D. (2017). Casein kinase 1-epsilon deletion increases mu opioid receptor-dependent behaviors and binge eating1. Genes Brain Behav. *16*, 725–738. https://doi.org/10.1111/gbb.12397.

STAR★METHODS

KEY RESOURCES TABLE

REAGENT or RESOURCE	SOURCE	IDENTIFIER
**Antibodies**		
Rabbit anti-c-Fos 9F6 mAb	Cell Signaling Technologies	Cat#2250; RRID:AB_2247211
Donkey anti-Rabbit IgG (H+L) Secondary Antibody Alexa Fluor 568	ThermoFisher Scientific	Cat#A10042; RRID:AB_2534017
**Bacterial and virus strains**		
Muribaculum intestinale YL7	Leibniz Institute German Collection of Microorganisms and Cell Cultures GmbH (DSMZ)	Cat#100746
Muribaculum intestinale YL27	Leibniz Institute German Collection of Microorganisms and Cell Cultures GmbH (DSMZ)	Cat#28989
Paramuribaculum intestinale B1404	Leibniz Institute German Collection of Microorganisms and Cell Cultures GmbH (DSMZ)	Cat#100764
Akkermansia muciniphila BAA-835	American Type Culture Collection (ATCC)	Cat#BAA-835
Lactobacillus johnsonii	This study	N/A
**Chemicals, peptides, and recombinant proteins**		
Ampicillin sodium salt	Patterson Veterinary	Cat#07-893-3819
Vancomycin hydrochloride	Almaject Inc.	Cat#72611-765-10
Neomycin sulfate	Fisher Scientific	Cat#BP266925
Metronidazole	Acros Organics	Cat#210340050
Sodium acetate	Millipore Sigma	Cat#S2889
Sodium propionate	Millipore Sigma	Cat#P5436
Sodium butyrate	Millipore Sigma	Cat#303410
**Critical commercial assays**		
Quick-DNA Fecal/Soil Microbe Miniprep Kit	Zymo Research	Cat#D6010
Quick-RNA Miniprep Kit	Zymo Research	Cat#R1055
MoBio PowerSoil DNA Isolation Kit	Qiagen	Cat#12888
PowerUp SYBR Green Master Mix	ThermoFisher Scientific	Cat#A25742
Apex Taq RED Master Mix	Genesee Scientific	Cat#42-138B
iScript cDNA Synthesis Kit	Bio-Rad	Cat#1708891
**Deposited data**		
Raw sequencing data from 16S profiling	This study	NCBI SRA: BioProject PRJNA789557
All data to reproduce figures	This study	CaltechDATA Repository: https://doi.org/10.22002/s8tfx-hwq49
**Experimental models: Organisms/strains**		
Mus musculus: C57BL/6J	The Jackson Laboratory	Cat#000664
Mus musculus: Gnotobiotic C57BL/6J	Caltech Gnotobiotic Facility	N/A
Mus musculus: B6.FVB-Tg(Npy-hrGFP)1Lowl/J	The Jackson Laboratory	Cat#006417
**Oligonucleotides**		
See Table S3	Caporaso et al.,[90] Lane,[91] Turner et al.,[92] Piper et al.,[93] and Reichenbac et al.[94]	N/A
**Software and algorithms**		
R (Version 3.6.3)	R Core Team[95]	https://www.r-project.org/
RStudio (Version 1.4.1106)	RStudio Team[96]	https://www.rstudio.com/
QIIME2 (Version 2019.10)	Bolyen et al.[97]	https://www.qiime2.org/

(Continued on next page)

Continued

REAGENT or RESOURCE	SOURCE	IDENTIFIER
Ethovision XT 10	Noldus et al.[98]	https://www.noldus.com/
Fiji/ImageJ (Version 1.53f51)	Schindelin et al.[99]	https://www.imagej.net/software/fiji/
MaAsLin2	Mallick et al.[100]	https://huttenhower.sph.harvard.edu/maaslin/
Chromas (2.6.6)	Technelysium	https://technelysium.com.au/wp/chromas/
BLAST	Altschul et al.[101]	https://blast.ncbi.nlm.nih.gov/Blast.cgi/
BORIS (7.13)	Friard and Gamba[102]	https://boris.unito.it/
GraphPad Prism (9.1.0)	GraphPad Software	https://www.graphpad.com/
Other		
Laboratory Autoclavable Rodent Diet	LabDiet	Cat#5010
High-Sucrose Purified Rodent Tablet (5TUL)	Test Diets	Cat#1811142
Calorie-Free Cellulose Reward Tablet (5TUW)	Test Diets	Cat#1812939
Custom Cellulose-Substituted Purified Rodent Tablet	Test Diets	N/A
Custom Sucralose-Substituted Purified Rodent Tablet	Test Diets	N/A
Custom AIN-76A 5 gram Purified Rodent Tablet	Test Diets	N/A
Rodent Diet with 60 kcal% Fat (High-Fat Diet)	Research Diets, Inc.	Cat#D12492
Chocolate Flavor Ensure®	Abbott Nutrition	Cat#53623
Bacto Brain Heart Infusion Broth	BD	Cat#237500
Chopped Meat Tubes	Anaerobe Systems	Cat#AS-811
Lactobacilli MRS Broth	BD	Cat#288130
Brucella Agar Plates with 5% Sheep's Blood	Teknova	Cat#B0150
Feeding Experimentation Device 2.0 (FED2)	Nguyen et al.[42]	https://hackaday.io/project/72964-feeding-experimentation-device-fed-20
Feeding Experimentation Device 3.0 (FED3)	Matikainen-Ankney et al.[58]	https://hackaday.io/project/106885-feeding-experimentation-device-3-fed3

RESOURCE AVAILABILITY

Lead contact

Further information and requests for resources and reagents should be directed to and will be fulfilled by the lead contact, Sarkis K. Mazmanian (sarkis@caltech.edu).

Materials availability

No new reagents were generated in this study.

Data and code availability

Microbial sequencing data have been deposited at the Sequence Read Archive (SRA: BioProject PRJNA789557) and are publicly available as of the date of publication. All other experimental data used to generate the figures reported in this paper can be found in the CaltechDATA Repository (CaltechDATA: https://doi.org/10.22002/s8tfx-hwq49), publicly available as of the date of publication.

This paper does not report original code.

Any additional information required to reanalyze the data reported in this paper is available from the lead contact upon request.

EXPERIMENTAL MODEL AND SUBJECT DETAILS

Mice

Wild-type C57BL/6J (The Jackson Laboratory, Cat#000664) and B6.FVB-Tg(Npy-hrGFP)1Lowl/J (The Jackson Laboratory, Cat#006417) mice were obtained from Jackson Laboratory at 8 weeks of age. Germ-free C57BL/6J mice were obtained from the Caltech gnotobiotic facility. All experiments were performed with male mice. Animals were group housed (2–5 mice per cage) unless otherwise specified. No statistical methods were used to predetermine sample size. For behavioral experiments, investigators were not blinded to treatment group unless otherwise specified.

Experimental mice were housed in sterilized microisolator cages and maintained on ad libitum autoclaved 5010 PicoLab Rodent Diet (LabDiet, Cat#5010) and sterilized water. Ambient temperature in the animal housing facilities was maintained at 21-24°C, 30-70% humidity, with a cycle of 13 hours light, 11 hours dark. All experiments were performed with approval from the Caltech Institutional Animal Care and Use Committee (IACUC).

Bacterial culture conditions

Akkermansia muciniphila (ATCC BAA-835), *Muribaculum intestinale* YL7 (DSM 100746), *Muribaculum intestinale* YL27 (DSM 28989), *Paramuribaculum intestinale* (DSM 100764), and the isolated strain of *L. johnsonii* were cultured, unshaken, under anaerobic conditions (10% CO_2, 10% H_2, 80% N_2) at 37°C. *M. intestinale* strains and *P. intestinale* were cultured in Chopped Meat Broth (Anaerobe Systems, Cat#AS-811), *A. muciniphila* was cultured in Bacto Brain Heart Infusion broth (BD Cat#237500), supplemented with 5 mg hemin and 0.1 mg menadione per liter, and the *L. johnsonii* isolate was cultured in BD Difco Lactobacilli MRS Broth (BD Cat#288130).

METHOD DETAILS

Antibiotic (ABX) treatment

Gut microbiota depletion with oral antibiotics (ABX) was performed by administration of ampicillin as sodium salt (1 g ampicillin/L, Patterson Veterinary, Cat#07-893-3819), vancomycin as hydrochloride salt (0.5 g vancomycin/L, Almaject Inc., Cat#72611-765-10), neomycin sulfate (1 g/L, Fisher Scientific, Cat#BP266925), and metronidazole (0.5 g/L, Acros Organics, Cat#210340050) to 8-week-old mice for a period of 4 weeks.[38,40] To encourage antibiotic uptake, ABX and VEH water was supplemented to 1% (w/v) with sucrose vehicle before filter sterilization (0.22 μm). Drinking water was replaced weekly. Administration of individual antibiotics was conducted using the same antibiotic concentrations and vehicle conditions. Animals removed from ABX for experimental reasons were switched to vehicle, which was maintained throughout behavioral testing.

For intragastric gavage administration of ABX, 200 μL of a filter-sterilized (0.22 μm) solution of ampicillin as sodium salt (15 mg ampicillin/mL), vancomycin as hydrochloride salt (7.5 mg vancomycin/mL), neomycin sulfate (15 mg/mL), and metronidazole (7.5 mg/mL) in water was administered once-daily to 8-week-old mice for a period of 10 days. Concentrated ABX solution was stored at 4°C for the treatment duration. Due to precipitation, ABX was briefly sonicated prior to daily gavage. VEH – i.g. animals were given an equal volume daily gavage of sterile water. The final ABX/VEH gavage occurred 2 hours prior to behavioral testing.

For subcutaneous administration of ABX, 200 uL of a filter-sterilized (0.22 μm) and sonicated solution of ampicillin as sodium salt (15 mg ampicillin/mL) and metronidazole (7.5 mg/mL) in saline were injected into the loose skin over the shoulders of the mouse 1 hour prior to behavioral testing. Neomycin and vancomycin were not included in the subcutaneous antibiotic cocktail as these antibiotics undergo negligible absorption when administered orally.[103] VEH – s.c. animals were given an equal-volume subcutaneous injection of saline.

Germ-free (GF) C57BL/6J mice were removed from gnotobiotic isolators under sterile conditions and transferred to sterilized microisolator cages 3 days prior to behavioral testing.

Microbiota transplant and microbial treatment

Fecal samples were collected from experimental mice, weighed, and resuspended in a 10-fold volume of sterile filtered (0.22 μm) reduced phosphate buffered saline (PBS) containing 1.5% (w/v) sodium bicarbonate under anaerobic conditions. The sample was mashed with a pipette tip to create a fecal slurry, which was centrifuged at 250 x g for 5 minutes to separate fecal solids. The bacterial supernatant was collected and 200 μL was administered by intragastric gavage to recipient mice. This procedure occurred once-daily for 3 days following antibiotic removal. Mice were rehoused in a new sterile cage on the first day receiving FMT. Non-FMT receiving control animals received gavages of reduced PBS with 1.5% (w/v) sodium bicarbonate. For autologous fecal microbiota transplants, mice from the same cage were assumed to share the same microbiota, and therefore samples from one cage would be collected and used for FMT in a given cage. Animals were given 2 weeks from the first day of FMT to allow for microbiota recovery prior to behavioral testing.

In the gut microbiota reconstitution experiment comparing the microbial diversity and bacterial loads between VEH, ABX+FMT, and ABX+SHAM mice, 3/4 ABX+SHAM mice in one cage were found dead during the 2-week recovery period between saline gavage and behavioral testing. We tentatively ascribe this phenomenon to opportunistic expansion of a gut pathobiont.

For experiments involving the supplementation of *A. muciniphila* or the mixture of S24-7 strains and *Lactobacillus johnsonii* (4-mix), autologous fecal bacterial supernatants were used to resuspend pelleted turbid bacterial cultures (2,400 x g for 20 minutes) for a final microbial density of 10^8-10^9 CFU/mL per microbe.[81] The S24-7 mixture consisted of equal bacterial culture volumes of *Muribaculum intestinale* YL7, *Muribaculum intestinale* YL27, and *Paramuribaculum intestinale* B1404. Resulting microbial suspensions were administered to recipient mice according to the same timeline as FMT administration.

Short-chain fatty acid treatment

SCFAs were administered as dissolved sodium salts (67.5 mM sodium acetate (Millipore Sigma, Cat#S2889), 25 mM sodium propionate (Millipore Sigma, Cat#P5436), 40 mM sodium butyrate (Millipore Sigma, Cat#303410)) in the drinking water before filter sterilization.[104,105] Control animals were placed on sodium- and pH-matched drinking water. Drinking water was replaced weekly and SCFA or sodium-matched treatment occurred throughout the entire course of ABX depletion and through behavioral testing.

Colony forming unit (CFU) analyses

Fecal material from mice was collected, weighed, and resuspended in a 10-fold volume of aerobic or anaerobic PBS in a sterile 1.5-mL microcentrifuge tube. Aerobic and anaerobic samples were briefly centrifuged at 250 x g for 5 minutes and the bacterial supernatants were serially diluted in aerobic or anaerobic PBS, respectively. Samples were plated in quadruplicate on both Brain Heart Infusion (BD, Cat#237500) agar supplemented with 5 mg hemin and 0.1 mg menadione per liter (BHIS), and Brucella agar plates supplemented with 5% (v/v) defibrinated sheep's blood (Teknova, Cat#B0150). Aerobic and anaerobic supernatants were cultured at 37°C aerobically and anaerobically, respectively, for 48 hours before counting of colony forming units. Plates where no colonies grew were given a measurement of 0 CFU/mg feces for purposes of statistical testing.

Analysis of fecal microbial load

Fecal samples were collected from experimental mice and immediately snap-frozen in liquid nitrogen before storage at -80°C. Samples were weighed and total fecal DNA was extracted using either a Qiagen PowerMag Soil DNA Isolation Kit (Qiagen, Cat#12888) or a Zymo Quick-DNA Fecal/Soil Microbe Miniprep Kit (Zymo Research, Cat#D6010), according to the manufacturer's protocol. DNA concentrations were determined via spectrophotometer. Extracted DNA was used as template for triplicate qPCR reactions (ThermoFisher Scientific, Cat#A25742) using universal bacterial primers (200 nM forward and reverse) against the microbial 16S rRNA (515F: 5'-GTGCCAGCMGCCGCGGTAA-3', 806R: 5'-GGACTACHVGGGTWTCTAAT-3').[90] qPCR signal was normalized to fecal DNA content and sample weight. See also Table S3.

Fecal microbiome community profiling

Bacterial 16S rRNA genes from extracted fecal DNA were PCR-amplified with barcoded primers targeting the V4 region. Sequencing was performed by either Microbiome Insights, Inc. (Vancouver, BC), or Laragen, Inc (Culver City, CA). For sequences prepared by Microbiome Insights, amplicons were sequenced with an Illumina MiSeq using the 300-bp paired-end kit (v.3) according to the protocol of Kozich et al.[106] For sequences prepared by Laragen, amplicons were sequenced according to the Earth Microbiome Protocol.[107] Sequences were analyzed using the QIIME2 (2019.10) software package.[97]

Demultiplexed reads were filtered for quality and denoised using the q2-deblur package. Sequences were trimmed to different lengths based on the quality scores of separate sequencing runs. These trim lengths are 147 bp, 220 bp, 151 bp, and 151 bp for the VEH/ABX+SHAM/ABX+FMT, individual antibiotic administration, SPF and autologous FMT, and microbial rescue experiments, respectively. Taxonomic classification of amplicon sequence variants (ASVs) was performed in QIIME2 using classify-sklearn with a classifier pre-trained on the Greengenes database (13_8 release). Phylogenetic diversity metrics were generated from ASV feature tables using q2-phylogeny and q2-diversity plugins. Within-subject diversity comparisons across multiple timepoints were generated using the q2-longitudinal plugin. Sampling depth was chosen based on manual analysis of the reads per sample in a given experiment. These sampling depths are 8836, 1147, 14785, and 22785 reads, for the VEH/ABX+SHAM/ABX+FMT, individual antibiotic administration (6/8 ABX and 4/8 Ampicillin samples did not meet rarefaction threshold), SPF and autologous FMT, and microbial rescue experiments, respectively. Weighted and unweighted UniFrac diversity metrics, alpha-diversity metrics, and Principal Coordinate Analyses (PCoAs) of diversity metrics were generated as implemented in QIIME2. Hypothesis tests for differences in diversity were tested using PERMANOVA as implemented in QIIME2. Differential abundance analysis of ASVs was performed using the MaAsLin2 R package to identify features that associate with treatment conditions.[100] In the individual antibiotic treatment experiment, the vancomycin condition was used as the reference level in a one-versus-all comparison. Differential abundance analysis based on relative abundances was restricted to the vancomycin condition given the significant reduction of microbial load in ABX and ampicillin-treated mice. For this analysis, cage was included as a random effect and treatment group was included as a fixed effect. Features were normalized by total sum scaling, and an FDR-corrected significance threshold was set at 0.1. Significant results from the differential abundance analysis are in Table S2.

Isolation of *Lactobacillus johnsonii*

Fecal samples from a mouse from the VEH experimental condition were collected and resuspended in a 10-fold volume of anaerobic Lactobacilli MRS Broth (BD, Cat#288130) in a sterile 10-mL microcentrifuge tube. Serial 10-fold dilutions in MRS broth were plated on MRS agar plates and cultured for 48 hours under anaerobic conditions (10% CO_2, 10% H_2, 80% N_2) at 37°C. Single colonies were re-streaked on MRS agar plates and colony PCR Sanger sequencing was conducted by Laragen, Inc. using universal 16S ribosomal RNA primers. *16S*: (27F: 5'- AGAGTTTGATCMTGGCTCAG-3', 1492R: 5'- GGTTACCTTGTTACGACTT-3').[91,92] Chromatogram traces were analyzed using Chromas (2.6.6) (Technelysium) and sequence alignment visualization to the differentially abundant *Lactobacillus* sp. ASV (100% match) was performed using EMBOSS Matcher.[108] The sequenced 16S rRNA was used as a query sequence for a BLASTN analysis (NCBI) and found to have >99.8% sequence identity to cultured strains of *Lactobacillus johnsonii*.[101] See also Table S3.

Hypothalamic neuropeptide expression

VEH and ABX animals were euthanized by cervical dislocation and hypothalami were extracted and snap-frozen in TRIzol reagent. Hypothalamic RNA was isolated using a Zymo Quick-RNA Miniprep Kit (Zymo Research, Cat#R1055) and reverse transcribed into cDNA using an iScript cDNA Synthesis Kit (Bio-Rad, Cat#1708891). Both steps were performed according to the manufacturer's directions. Triplicate qPCR reactions (ThermoFisher Scientific, Cat#A25742) were run using the following primer

sets: *AgRP*: (5'-TGCTACTGCCGCTTCTTCAA-3' and 5'-CTTTGCCCAAACAACATCCA-3'), *NPY*: (5'-TAACAAGCGAATGGGGCTGT-3' and 5'-ATCTGGCCATGTCCTCTGCT-3'), *POMC*: (5'-AGGCCTGACACGTGGAAGAT and 5'-AGGCACCAGCTCCACACAT-3').[93] qPCR signal was normalized to the expression of the 18S eukaryotic rRNA *18S*: (5'-TTCCGATAACGAACGAGACTCT-3' and 5'-TGG CTGAACGCCACTTGTC-3').[94] See also Table S3.

Brain sample collection

For c-Fos analysis of *NPY*+ neurons in the ARC, VEH, ABX, and overnight fasted mice were taken from their home cages and euthanized. For c-Fos analysis of reward-related brain regions, single-housed VEH and ABX mice were euthanized 60 minutes after the introduction of either an empty glass dish (-Stimulus) or a glass dish containing approximately 2 grams of high-sucrose pellets (5TUL, Test Diets, Cat#1811142) (+Stimulus) into their home cage. Treatment groups were given prior exposure to the glass dish and previously acclimated to the high-sucrose pellets (100 mg provided in the home cage the day before) to reduce effects of novelty. Euthanasia was conducted via a 150 μL intraperitoneal injection of a 1:10 saline dilution of Euthasol (Virbac, Cat#PVS111), a solution of sodium pentobarbital and sodium phenytoin. Mice were transcardially perfused with chilled PBS followed by chilled 4% paraformaldehyde in PBS. Brains were harvested and stored in 4% paraformaldehyde in PBS for 2 days at 4°C before transfer to a solution of 0.02% sodium azide in PBS at 4°C prior to sectioning.

Brain sectioning and c-Fos measurement

Brains were embedded in 2% (w/v) UltraPure low melting point agarose (ThermoFisher Scientific, Cat#16520100) in PBS containing 0.02% sodium azide and 50-μm-thick coronal sections were sectioned using a vibratome (Leica Biosystems, Cat#VT1000). Every third slice was collected and stored at 4°C in 0.02% sodium azide in PBS until staining. Coronal brain sections were incubated with primary antibody (1:500 rabbit anti-cFos (9F6), CST Cat#2250) in blocking buffer (10% horse serum, 0.3% Triton X-100, 0.02% sodium azide in PBS) and placed on a benchtop rocker overnight at room temperature. Primary antibody-stained slices underwent three 45-minute room temperature washes in PBS containing 0.3% Triton X-100. Secondary antibody (1:1000 donkey anti-rabbit Alexa Fluor 568, ThermoFisher Scientific, Cat#A10042) in blocking buffer was incubated with the washed slices for 2 hours rocking at room temperature protected from light. Slices underwent three washes, 2 hours each, in sterile PBS at room temperature, before mounting on Superfrost Plus microscope slides (Fisher Scientific, Cat#12-550-15). Slices were drained of excess liquid using a Kimwipe (Fisher Scientific) and Prolong Diamond Antifade Mountant with DAPI (ThermoFisher Scientific, Cat#P36962) was used to adhere the coverslip. Slides were left at room temperature overnight protected from light to solidify the mountant prior to imaging.

Microscopic imaging and cell quantification

Imaging was performed using a Zeiss LSM 880 confocal laser scanning microscope using Zen software. All images shown and quantified are maximum intensity projections in the z-direction of z-stacks of mounted 50-μm-thick coronal slices using a 10X objective lens. Tile-scans with stitching were employed to capture brain regions larger than a single field of view. c-Fos+ and *NPY*+ cell bodies unambiguously brighter than background signal were quantified manually in Fiji/ImageJ (1.53f51) by a researcher blinded to treatment group and stimulus status. All images were minimally processed for brightness and contrast. Regions of interest (ROI) for quantification of cell density were defined using anatomical landmarks. For each mouse, the slice with greatest correspondence to the anterior-posterior coordinates of the target brain region was used for quantification.

Anterior-posterior coordinates for imaging were +1.0 mm to +1.3 mm (nucleus accumbens, dorsal striatum), -1.7 to -1.3 mm (lateral hypothalamus), -1.8 to -1.5 (arcuate nucleus of the hypothalamus) -3.7 to -3.4 mm (ventral tegmental area), -1.2 to -0.9 mm (basolateral amygdala).[109]

Free-feeding intake of high-sucrose pellets

Experimental mice were single-housed the day prior to behavioral testing. To reduce effects of neophobia, mice were housed overnight with an automated pellet dispenser[42] (Feeding Experimentation Device 2.0, (FED2)) in the "off" state and acclimated to 100 mg (five pellets) of the high-sucrose pellets (5TUL, Test Diets, Cat#1811142) in their home cage. This palatable food is a pelleted formulation of the widely studied AIN-76A complete diet, frequently used as a high-sugar stimulus for binge behavior in rodent studies, and is reported to be preferred at a ratio of >9:1 compared to standard chow in food choice assays.[19,69,110–112] The morning of behavioral testing, cages were manually checked to ensure the five acclimation pellets were consumed. Mice were provided ad lib chow and treatment water during the acclimation period. The unfasted mice, FED2, and treatment water were moved into a new cage without chow and the FED2 was stocked with high-sucrose pellets and placed in the "on" state. Mice were left to consume pellets from the FED2, which records occurrences of pellet retrieval events to an internal memory card, for at least 2 hours or until 50 pellets had been retrieved. In the sucralose substitution experiment, the sucrose in the high-sucrose pellets was replaced with a mixture of microcrystalline cellulose and sucralose for a final diet concentration of 48% (w/w) cellulose and 2% (w/w) sucralose (Test Diets). Intake rate was modeled using RStudio running R (3.6.3) by fitting an exponential function[25] with a fixed y-intercept of zero to each trace of cumulative 2-hour intake. The derivative of this function was taken and calculated at each minute.

To confirm that the FED2s provided an accurate representation of pellet consumption, a cohort of VEH and ABX mice (n=5/group) were tested for free-feeding intake as above but with simultaneous video recording during the 2-hour test session. Pellet consumption events over the 2-hour test session were manually recorded using BORIS[102] (7.13) by an experienced researcher blinded to animal treatment status. The average FED accuracy, calculated as (Pellets Eaten / FED Retrieval Events) of all tested mice was

1.00 when rounded to the nearest hundredth place (mean=1.0019, standard deviation=0.0154). On five occasions, representing ~1% of all recorded retrieval events for this cohort (5/493, 3 VEH and 2 ABX), the FED2s were observed to dispense two pellets instead of one, in line with the previously reported accuracy of the FED2s,[42] and enabling values of FED accuracy >1. On four occasions, representing <1% of all recorded retrieval events for this cohort (4/493, 2 VEH and 2 ABX), mice were observed to retrieve a pellet from the FED2 without having consumed it by the end of the 2-hour session. To test if the comparisons of time-series pellet retrieval data could be considered representative of time-series pellet consumption data, we performed within-mouse Kolmogorov-Smirnov comparisons (R, 3.6.3) of cumulative probability distributions of FED-recorded retrieval events and manually determined pellet consumption events. In all mice (10/10), the p-value was >0.995 and the KS statistics were <0.08 (mean = 0.0478, standard deviation = 0.0187), suggesting that FED-based pellet retrieval metrics are reflective of consumption events. Full information regarding comparisons to pellet consumption data can be found in Table S1.

Free-feeding intake of a high-fat diet

Experimental mice were single-housed the day prior to behavioral testing. To reduce effects of neophobia, mice were housed with 200 mg of the high-fat diet (HFD) (Research Diets, Cat#D12492) provided in a glass dish. The HFD chosen has previously been shown to induce binge-like consumption responses in mice.[55] The morning of behavioral testing, cages were manually checked to ensure the 200 mg of HFD was consumed. Mice were provided ad lib chow and treatment water during the acclimation period. The unfasted mice and treatment water were moved into a new cage without chow containing a pre-weighed amount of HFD (~2 g). Mice were free to consume for 2 hours. Every 30 minutes, the HFD was briefly removed from the cage, weighed, and returned to the cage. The difference in HFD weight from baseline was taken as intake.

Free-feeding intake of Ensure®

Experimental mice were single-housed the day prior to behavioral testing. To reduce effects of neophobia, mice were housed with 1 mL of chocolate Ensure® (Abbott Nutrition, Cat#53623) provided in a glass dish. Ensure® is a palatable liquid diet known to induce binge-like consumption responses in mice.[56] The morning of behavioral testing, cages were manually checked to confirm the 1 mL of Ensure® was consumed. Mice were provided ad lib chow and treatment water during the acclimation period. The unfasted mice and treatment water were moved into a new cage without chow containing an Ensure®-filled graduated 10-mL pipet outfitted with a ball-bearing sipper tube to allow for controlled consumption.[113] The end of the ball-bearing sipper was primed with chocolate Ensure® to assist in induction of consumption. Mice were free to consume for 2 hours and recorded with a video camera. Recordings were analyzed for drinking activity using BORIS by an experienced researcher blinded to treatment condition. The levels of Ensure® were measured visually at 30-minute timepoints. Differences in Ensure® volume from baseline were taken as intake. Cages were visually examined after the experiment for any evidence of Ensure® leakage and no leakage was observed.

Bout structure analyses

For measurements of FED bout length, pellets per bout, and number of bouts, a feeding bout was defined as at least two pellet retrieval events occurring within 60 seconds of each other.[58,114] A bout was considered terminated when at least 60 seconds have passed between pellet retrieval events.[58] Analysis of time-series data was performed in RStudio running R (3.6.3).

For measurements of Ensure® drinking bout length, drinking events within 60 seconds of one another were defined as being within the same bout.[115]

Operant conditioning

Custom-built acrylic arenas (10”x10”x12”) equipped with an operant conditioning unit with programmable active and inactive nose-poke ports (FED3) were used for fixed-ratio 1 (FR1) training and progressive ratio (PR) testing.[28,58] The assignment of active and inactive ports was reversed for half of the mice within a given treatment group to account for potential arena effects. Experimental mice were single-housed and acclimated to 100 mg of high-sucrose pellets the day prior to beginning FR1 training. During FR1 training, mice were placed on a restricted feeding schedule of 2–3 g chow/day, given as a single bolus after the daily training session, to maintain ~95% of their starting body weight. FR1 training sessions, where a single nose-poke in the active port resulted in the delivery of a single high-sucrose pellet, lasted for 60 minutes and proceeded for at least 7 days. Mice unable to reach the training criterion, defined as 3 consecutive days of ≥75% correct port discrimination with at least 20 pellets retrieved, were trained up to a maximum of 7 additional days, or until the criterion was met. 12/12 ABX mice and 10/12 VEH mice successfully reached the training criteria.

Successfully trained mice were returned to an ad libitum feeding schedule for 3 days before proceeding to PR testing. Mice underwent daily 90-minute PR breakpoint sessions, where the number of active pokes, N, required to obtain pellet n+1 is increased after each successful pellet retrieval event, based on the formula $N_{n+1} = 5e^{(0.2n)} - 5$, rounded to the nearest integer.[116] The breakpoint is defined as the final ratio completed by the mouse in the PR session. Unfasted animals were tested daily until breakpoints were considered stabilized (either within ±10% variance in the number of pellets obtained or ±1 pellet when <10 pellets are obtained, over 3 consecutive days).[28,117] Performance in the final PR assay was taken as the breakpoint for comparisons between treatment groups.

Brief-access dietary selection assay

Unfasted experimental mice were habituated to the testing chamber (an empty cage with no bedding) for 10 minutes prior to the introduction of a bolus of pre-weighed edible stimuli in a glass dish—either standard chow, pure microcrystalline cellulose pellets (5TUW,

Test Diets, Cat#1812939), a custom formulation of the high-sucrose pellets in which the sucrose was replaced by microcrystalline cellulose (Test Diets), or the high-sucrose pellets used throughout the study (5TUL, Test Diets, Cat#1811142). The stimuli were tested in the order listed. Animals were left to freely consume the stimulus for 10 minutes and the difference in stimulus weight was measured as intake. To reduce neophobia, the day prior to testing the mice were given a small amount of each edible stimulus in their home cage (100 mg/mouse) and housed with a glass dish overnight. The morning of behavioral testing, cages were checked to ensure the acclimation stimulus was consumed. Mice were given 1 day of rest between assays.

High-sucrose pellet binge-like consumption assay

To reduce anxiety and neophobia, animals were habituated to the testing chamber (an empty cage with no bedding) for 1 hour and given free access to ~1 gram of the high-sucrose pellets (5TUL, Test Diets, Cat#1811142) in a glass dish.[70,118] This habituation step occurred prior to antibiotic removal to reduce potential environmental contamination of antibiotic-treated mice.

For behavioral testing, habituated mice were placed in the testing chamber for 10 minutes prior to being given a pre-weighed bolus of approximately 1.5 grams of high-sucrose pellets in a glass dish and allowed to consume freely for 1 hour. The uneaten pellets were weighed, and the difference was recorded.

Fasting-refeeding assays

Single-housed experimental mice were fasted overnight for 16 hours with access to treatment water. To reduce neophobia, the day prior to fasting, animals refed with high-sucrose pellets were given access to 100 mg (five pellets) of high-sucrose pellets, and during the fast, were housed with a FED2 in the "off" state. For chow refeeding, a pre-weighed amount of chow was returned to the cage and weighed at 30-minute intervals. The difference in chow weight over time was taken as intake. For refeeding with the high-sucrose pellets, the FED2 was stocked with pellets and placed into the "on" state.

Homeostatic food intake measurements

Experimental mice were single-housed and provided with ad lib chow for the duration of VEH/ABX treatment. The difference in chow weight was measured and taken as intake.

For home-cage AIN-76A intake, custom-manufactured 5-gram tablets of AIN-76A were provided as the exclusive food source to VEH/ABX mice for 1 week, starting after 4 weeks of VEH/ABX. The difference in AIN-76A weight compared to baseline was measured and taken as intake.

Baited and unbaited hole board assay

Experimental mice were placed in a hole board apparatus (40 cm x 40 cm x 35 cm, 3 cm hole diameter, Stoelting, Cat#62015) with 16 holes arranged in a 4 x 4 grid and left to explore for 10 minutes. In the unbaited assay, the holes were empty, mice were video recorded, and the number of spontaneous head dips was measured by a researcher blinded to treatment group as a metric for exploratory behavior. In the stimulus-baited assay, each apparatus hole was baited with a single high-sucrose pellet, and the number of pellets consumed after 10 minutes was used as a readout.[46]

Anxiety assays

Open field assays were conducted in white acrylic arenas (50 x 50 x 30 cm). Mice were recorded via an overhead camera for 10 minutes. The time spent in the center (30 x 30 cm) zone was measured and quantified using Ethovision XT 10 (Noldus Information Technology).

Elevated plus maze assays were conducted on a white EPM apparatus (28-cm arm length, 9 x 9 cm center zone) with black acrylic walls (16 cm). Mice were placed in the center and recorded via an overhead camera for 5 minutes. The time spent in the exposed open arms was measured and quantified using Ethovision XT 10.

QUANTIFICATION AND STATISTICAL ANALYSIS

Statistical tests and data visualization were performed in GraphPad Prism (9.1.0), QIIME2 (2019.10),[97] and RStudio[96] running R (3.6.3)[95] using the ggplot2 package. Statistical tests and replicate numbers are indicated in the respective figure legends and exact p-values for all comparisons made are reported in Table S1. Significantly different features as detected by MaAsLin2 analysis[100] are reported in Table S2. Error bars represent the standard error of the mean. For box-and-whisker plots, the whiskers represent the minimum and maximum values, the box extends from the 25th to the 75th percentile of the data, and the line within the box denotes the median. ****$p<0.0001$, ***$p<0.001$, **$p<0.01$, *$p<0.05$, ns: not significant.

Current Biology, Volume *33*

Supplemental Information

Gut microbiota suppress feeding induced by palatable foods

James Ousey, Joseph C. Boktor, and Sarkis K. Mazmanian

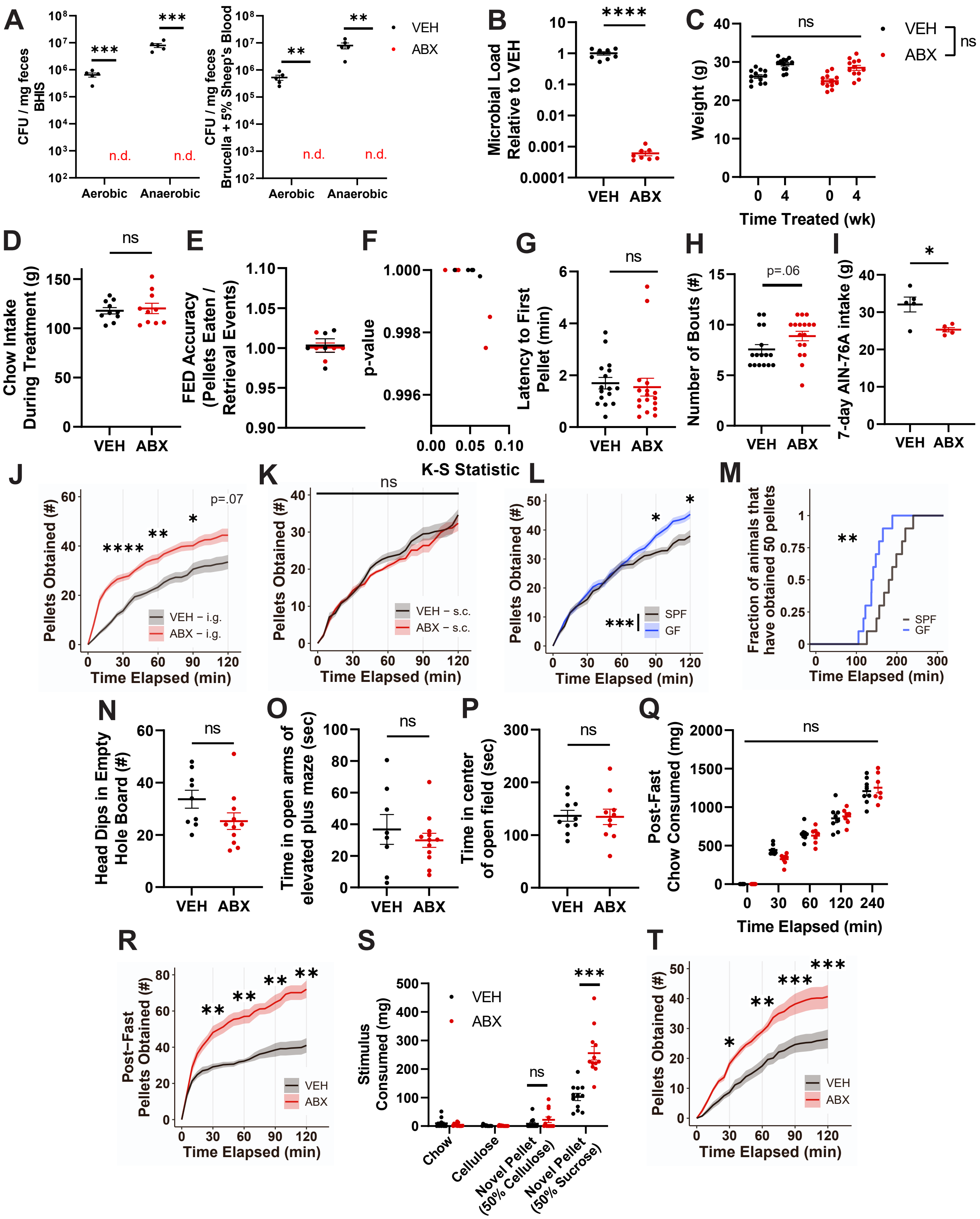
A
CFU / mg feces BHIS
Aerobic
Anaerobic
n.d.
CFU / mg feces Brucella + 5% Sheep's Blood
VEH
ABX
B
Microbial Load Relative to VEH
C
Weight (g)
Time Treated (wk)
ns
D
Chow Intake During Treatment (g)
E
FED Accuracy (Pellets Eaten / Retrieval Events)
F
p-value
K-S Statistic
G
Latency to First Pellet (min)
H
Number of Bouts (#)
p=.06
I
7-day AIN-76A intake (g)
J
Pellets Obtained (#)
Time Elapsed (min)
p=.07
VEH – i.g.
ABX – i.g.
K
VEH – s.c.
ABX – s.c.
L
SPF
GF
M
Fraction of animals that have obtained 50 pellets
N
Head Dips in Empty Hole Board (#)
O
Time in open arms of elevated plus maze (sec)
P
Time in center of open field (sec)
Q
Post-Fast Chow Consumed (mg)
R
Post–Fast Pellets Obtained (#)
S
Stimulus Consumed (mg)
Chow
Cellulose
Novel Pellet (50% Cellulose)
Novel Pellet (50% Sucrose)
T

Figure S1. Physiological and behavioral measurements of microbiota-depleted mice reveal effects on food intake are robust and depend on dietary composition, Related to Figure 1.

(A) Quantification of aerobic and anaerobic microbial growth via colony-forming-unit analysis from fecal samples of VEH and ABX mice (n=5/group) on non-selective media after 4 weeks of treatment. n.d.: not detected. Shown is the mean (± SEM). Significance calculated via two-tailed Student's t-tests.

(B) Microbial load in fecal samples relative to VEH mice as measured by qPCR with universal bacterial primers. Shown is the mean (± SEM). Significance calculated via two-tailed Student's t-test (n=8/group).

(C) Body weight of VEH and ABX-treated animals (n=12/group) immediately prior to and after 4 weeks of treatment. Shown is the mean (± SEM). Treatment effect and Time × Treatment interaction effect significance calculated via two-way repeated measures ANOVA.

(D) Chow intake of single-housed VEH and ABX (n=10/group) mice over 4 weeks of treatment. Shown is the mean (± SEM). Significance calculated via two-tailed Student's t-test.

(E) Quantification of FED reflection of consumption behavior in VEH and ABX mice (n=5/group) determined via comparison of FED-recorded high-sucrose pellet retrieval events to manual video analysis of pellets consumed during 2 hours of free access. Shown is the mean (± SEM) for both treatment groups.

(F) K-S statistics and p-values of within-subject comparisons of cumulative distribution functions of FED-recorded pellet retrieval events and manually recorded pellet consumption events in VEH and ABX mice (n=5/group).

(G) Latency to retrieve the first pellet in VEH (n=16) and ABX (n=17) mice. Shown is the mean (± SEM). Significance calculated via two-tailed Student's t-test.

(H) Cumulative feeding bouts over the first 2 hours of high-sucrose pellet exposure in VEH (n=16) and ABX (n=17) mice. Shown is the mean (± SEM). Significance calculated via two-tailed Student's t-test.

(I) Home cage intake of high-sucrose AIN-76A tablets in single-housed VEH and ABX (n=5/group) mice over 1 week. Shown is the mean (± SEM). Significance calculated via two-tailed Student's t-test.

(J) Cumulative retrieval of high-sucrose pellets between VEH-gavage (VEH – i.g., n=10) and ABX-gavage (ABX – i.g., n=8) mice. Shown is the mean (± SEM) plotted every 5 minutes. Significance calculated via two-way repeated measures ANOVA using 30-minute timepoints followed by Šidák's multiple comparisons test.

(K) Cumulative retrieval of high-sucrose pellets between VEH-subcutaneous (VEH – s.c.) and ABX-subcutaneous (ABX – s.c.) mice (n=8/group). Shown is the mean (± SEM) plotted every 5 minutes. Time × Treatment interaction effect significance calculated via two-way repeated measures ANOVA using 30-minute timepoints.

(L) Cumulative retrieval of high-sucrose pellets between SPF and germ-free (GF) mice (n=10/group). Shown is the mean (± SEM) plotted every 5 minutes. Timepoint and Time × Treatment interaction effect significance calculated via two-way repeated measures ANOVA using 30-minute timepoints followed by Šidák's multiple comparisons test.

(M) Empirical cumulative distribution plot of mice in SPF and GF cohorts that have retrieved 50 high-sucrose pellets (1 gram). Significance calculated via Mann-Whitney U test.

(N) Spontaneous exploratory head dips from VEH (n=9) and ABX (n=11) mice in a 10-minute empty hole-board assay. Shown is the mean (± SEM). Significance calculated via two-tailed Student's t-test.

(O) Open-arm time measurements for VEH (n=8) and ABX (n=12) in an elevated plus maze assay for anxiety. Shown is the mean (± SEM). Significance calculated via two-tailed Student's t-test.

(P) Exposed center time measurements for VEH and ABX (n=10/group) in an open field assay for anxiety. Shown is the mean (± SEM). Significance calculated via two-tailed Student's t-test.
(Q) Fasting-refeeding measurements of fasted VEH (n=8) and ABX (n=7) mice when refed with standard chow. Shown is the mean (± SEM). Time × Treatment interaction effect significance calculated via two-way repeated measures ANOVA.
(R) Fasting-refeeding pellet retrieval measurements of fasted VEH (n=6) and ABX (n=7) mice when refed with high-sucrose pellets. Shown is the mean (± SEM) plotted every 5 minutes. Significance calculated via two-way repeated measures ANOVA using 30-minute timepoints followed by Šidák's multiple comparisons test.
(S) Intake during 10-minute brief-access assays of different edible stimuli presented to VEH and ABX treated mice (n=12/group). Shown is the mean (± SEM). Significance calculated via two-way repeated measures ANOVA followed by Šidák's multiple comparisons test.
(T) Cumulative retrieval of 2% (w/w) sucralose pellets in VEH (n=8) and ABX (n=6) mice. Shown is the mean (± SEM) plotted every 5 minutes. Significance calculated via two-way repeated measures ANOVA using 30-minute timepoints followed by Šidák's multiple comparisons test.
****$p<0.0001$, ***$p<0.001$, **$p<0.01$, *$p<0.05$, ns: not significant.

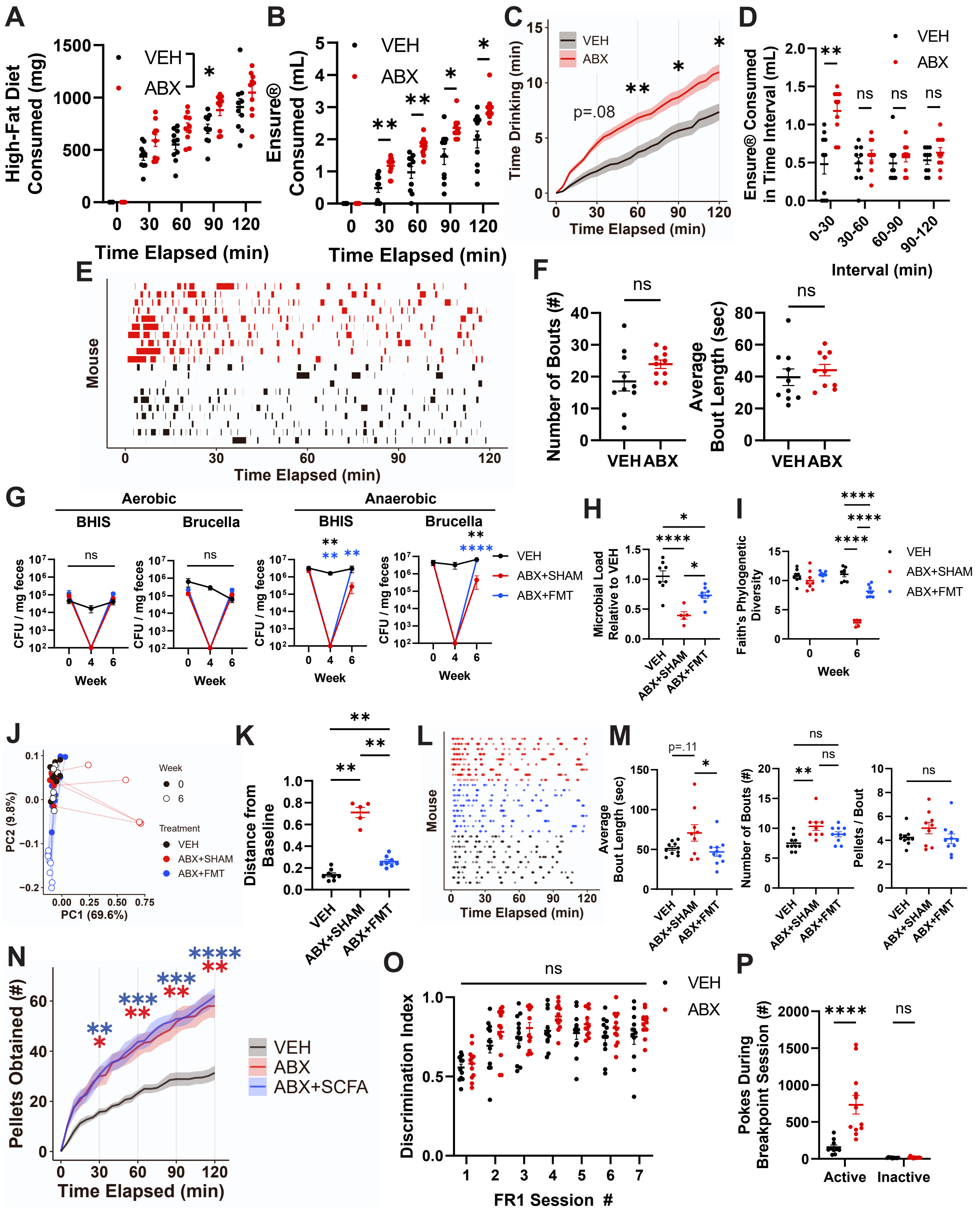

A
High-Fat Diet Consumed (mg)
VEH
ABX
Time Elapsed (min)
B
Ensure® Consumed (mL)
VEH
ABX
Time Elapsed (min)
C
Time Drinking (min)
VEH
ABX
p=.08
Time Elapsed (min)
D
Ensure® Consumed in Time Interval (mL)
VEH
ABX
ns
0-30
30-60
60-90
90-120
Interval (min)
E
Mouse
Time Elapsed (min)
F
Number of Bouts (#)
Average Bout Length (sec)
ns
VEH
ABX
G
Aerobic
Anaerobic
BHIS
Brucella
CFU / mg feces
Week
ns
VEH
ABX+SHAM
ABX+FMT
H
Microbial Load Relative to VEH
VEH
ABX+SHAM
ABX+FMT
I
Faith's Phylogenetic Diversity
Week
VEH
ABX+SHAM
ABX+FMT
J
PC2 (9.8%)
PC1 (69.6%)
Week
Treatment
VEH
ABX+SHAM
ABX+FMT
K
Distance from Baseline
VEH
ABX+SHAM
ABX+FMT
L
Mouse
Time Elapsed (min)
M
p=.11
Average Bout Length (sec)
Number of Bouts (#)
Pellets / Bout
ns
VEH
ABX+SHAM
ABX+FMT
N
Pellets Obtained (#)
VEH
ABX
ABX+SCFA
Time Elapsed (min)
O
Discrimination Index
ns
VEH
ABX
FR1 Session #
P
Pokes During Breakpoint Session (#)
ns
Active
Inactive

Figure S2. Additional behavioral parameters from free-feeding and operant conditioning assays and microbiome diversity measurements of FMT recipient mice, Related to Figures 1 and 2

(A) Cumulative consumption of a high-fat diet in VEH and ABX mice (n=10/group). Shown is the mean (± SEM). Treatment effect significance calculated via two-way repeated measures ANOVA at 30-minute timepoints followed by Šidák's multiple comparisons test.
(B) Cumulative consumption of Ensure® in VEH and ABX mice (n=10/group). Shown is the mean (± SEM). Significance calculated via two-way repeated measures ANOVA at 30-minute timepoints followed by Šidák's multiple comparisons test.
(C) Cumulative time drinking Ensure® in VEH and ABX mice (n=10/group). Shown is the mean (± SEM). Significance calculated via two-way repeated measures ANOVA at 30-minute timepoints followed by Šidák's multiple comparisons test.
(D) Consumption of Ensure® in VEH and ABX mice (n=10/group) across 30-minute intervals. Shown is the mean (± SEM). Significance calculated via two-way repeated measures ANOVA followed by Šidák's multiple comparisons test.
(E) Raster plot of Ensure® drinking bouts in VEH and ABX mice.
(F) Bout structure analyses of Ensure ® drinking bouts over 2 hours of access. Shown is the mean (± SEM). Significance calculated via two-tailed Student's t-test.
(G) Quantification of aerobic and anaerobic microbial growth via colony-forming-unit analysis from fecal samples of VEH, ABX+SHAM, and ABX+FMT mice ((Week 6, ABX+SHAM n=5), n=8/group all others) on non-selective media over the course of treatment. Shown is the mean (± SEM). Significance calculated via mixed-effects modeling followed by Tukey's multiple comparisons test (within timepoints). Black and blue asterisks denote ABX+SHAM vs. VEH and ABX+SHAM vs. ABX+FMT comparison significance, respectively.
(H) Microbial load in fecal samples of ABX+SHAM (n=5) and ABX+FMT (n=8) mice relative to VEH (n=8) mice at 6 weeks of treatment (2 weeks post-FMT) as measured by qPCR with universal bacterial primers. Shown is the mean (± SEM). Significance calculated via one-way ANOVA followed by Tukey's multiple comparisons test.
(I) Faith's Phylogenetic Diversity in VEH (n=8), ABX+SHAM (Week 0 n=8, Week 6 n=5), and ABX+FMT (n=8) mice at baseline and 6 weeks of treatment. Shown is the mean (± SEM). Significance calculated via mixed-effects modeling followed by Tukey's multiple comparisons test (within timepoints).
(J) PCoA of weighted UniFrac distances in VEH (n=8), ABX+SHAM (Week 0 n=8, Week 6 n=5), and ABX+FMT (n=8) mice at baseline and at 6 weeks of treatment.
(K) Within-subject weighted UniFrac distances in VEH (n=8), ABX+SHAM (n=5), and ABX+FMT (n=8) mice at 6 weeks of treatment compared to baseline. Shown is the mean (± SEM). Significance calculated via one-way ANOVA Kruskal-Wallis of within-subject distance comparisons to baseline followed by Mann-Whitney U tests subject to FDR correction as implemented in QIIME2. See also Table S1.
(L) Raster plot of pellet retrieval events of mice in VEH, ABX+SHAM, and ABX+FMT cohorts.
(M) Microstructure analyses of pellet retrieval events of mice in VEH, ABX+SHAM, and ABX+FMT cohorts over the first 2 hours of high-sucrose pellet exposure. Shown is the mean (± SEM). Significance calculated via one-way ANOVA followed by Tukey's multiple comparisons test.
(N) Cumulative retrieval of high-sucrose pellets of VEH, ABX, and ABX+SCFA (n=8/group) mice. Shown is the mean (± SEM) plotted every 5 minutes. Significance calculated via two-way repeated measures ANOVA using 30-minute timepoints followed by Tukey's multiple comparisons test (within timepoints). Red and blue asterisks denote ABX vs. VEH and ABX+SCFA vs. VEH comparison significance, respectively.

(O) Discrimination Indices (active pokes/total pokes) between VEH and ABX (n=12/group) mice during the first 7 days of FR1 training sessions. Shown is the mean (± SEM). Session × Treatment interaction effect significance calculated via two-way repeated measures ANOVA.
(P) Total active and inactive pokes in VEH (n=10) and ABX (n=12) mice during the progressive ratio breakpoint session. Shown is the mean (± SEM). Significance calculated via two-way repeated measures ANOVA followed by Šidák's multiple comparisons test.

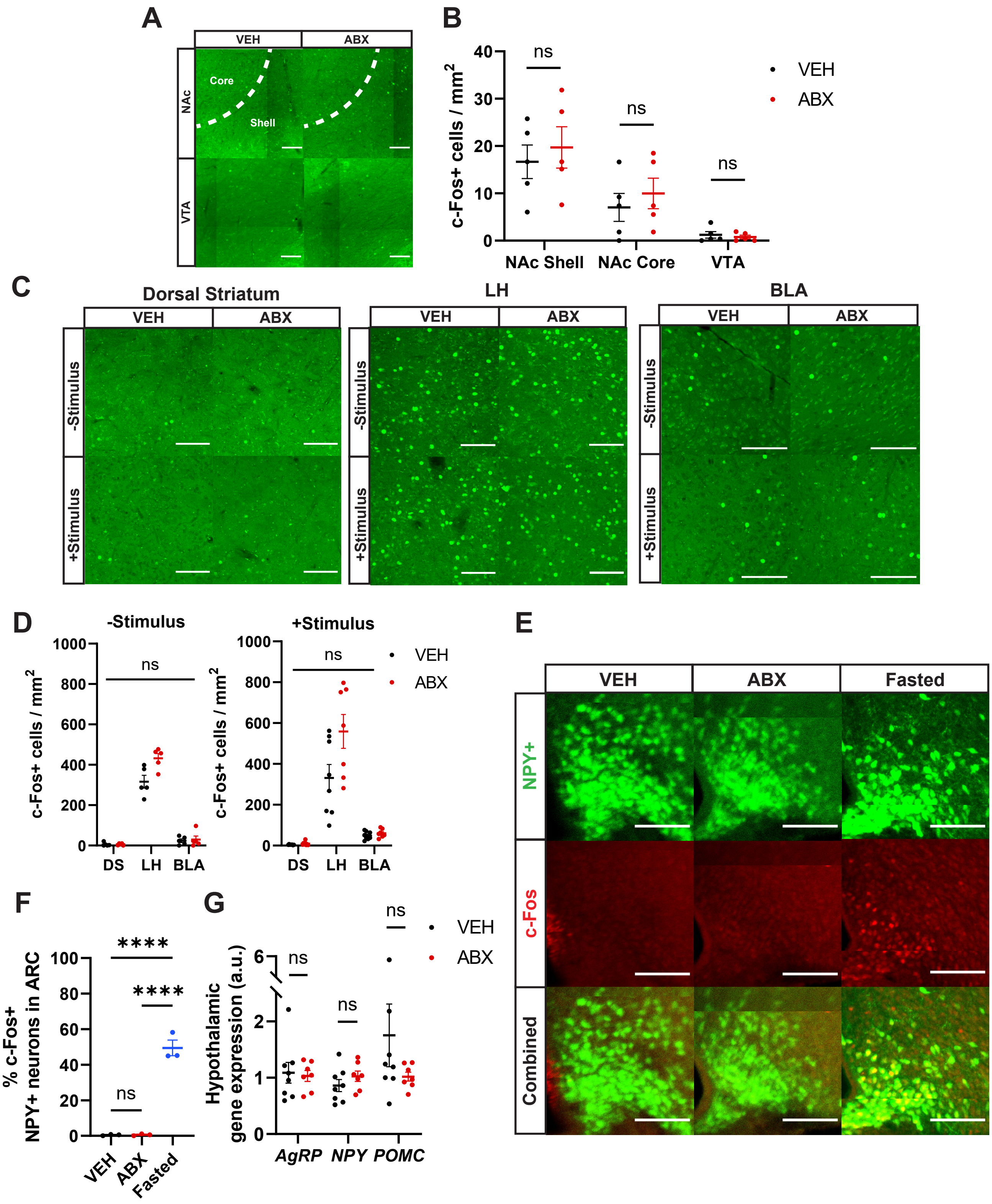

A
VEH
ABX
NAc
VTA
Core
Shell
B
c-Fos+ cells / mm2
40
30
20
10
0
ns
ns
ns
NAc Shell
NAc Core
VTA
VEH
ABX
C
Dorsal Striatum
LH
BLA
VEH
ABX
-Stimulus
+Stimulus
D
-Stimulus
+Stimulus
c-Fos+ cells / mm2
1000
800
600
400
200
0
ns
DS
LH
BLA
VEH
ABX
E
VEH
ABX
Fasted
NPY+
c-Fos
Combined
F
% c-Fos+ NPY+ neurons in ARC
100
80
60
40
20
0
****
****
ns
VEH
ABX
Fasted
G
Hypothalamic gene expression (a.u.)
6
2
1
0
ns
ns
ns
AgRP
NPY
POMC
VEH
ABX

Figure S3. High-sucrose pellet-induced neural activity in reward-related brain regions and characterization of homeostatic feeding signals in VEH and ABX mice, Related to Figure 2

(A) Representative images of the nucleus accumbens (NAc) and ventral tegmental area (VTA) in VEH and ABX mice without access to high-sucrose pellets, with c-Fos intensity represented in green. Scale bar is 100 microns. Images are cropped to emphasize the region of interest.

(B) Density of c-Fos+ neurons in the NAc shell, NAc core, and VTA (n=5/group) without access to high-sucrose pellets. Shown is the mean (± SEM). Significance calculated via two-way ANOVA with microbiota status and access to high-sucrose pellets as factors, followed by Bonferroni's multiple comparisons test (within brain regions). Data for mice given access to high-sucrose pellets is shown in Figures 2D and 2E.

(C) Representative images of c-Fos+ neurons in the dorsal striatum, lateral hypothalamus (LH), and basolateral amygdala (BLA) with (+Stimulus) and without (-Stimulus) 1 hour of access to high-sucrose pellets. Images have been cropped to aid cell body visualization. Scale bar is 100 microns.

(D) Density of c-Fos+ neurons in the dorsal striatum, LH, and BLA with (dorsal striatum: n=8/group, LH and BLA: n=8 VEH, n=7 ABX) and without (all regions: n=5/group) 1 hour of access to high-sucrose pellets. Shown is the mean (± SEM). Within-brain region Treatment × Stimulus interaction significance calculated via two-way ANOVA with microbiota status and access to high-sucrose pellets as factors.

(E–F) Representative images (E) and quantification (F) of c-Fos+ NPY+ neurons in the arcuate nucleus of the hypothalamus of VEH, ABX, or fasted (n=3/group) *NPY-hrGFP* transgenic mice. Shown is the mean (± SEM). Significance calculated via one-way ANOVA followed by Tukey's multiple comparisons test. Images have been cropped to aid cell body visualization. Scale bar is 100 microns.

(G) Hypothalamic neuropeptide expression as measured by qPCR in VEH (n=8) and ABX (n=7) mice. Shown is the mean (± SEM). Significance calculated via two-tailed Student's t-test followed by Holm-Šidák's correction for multiple comparisons.

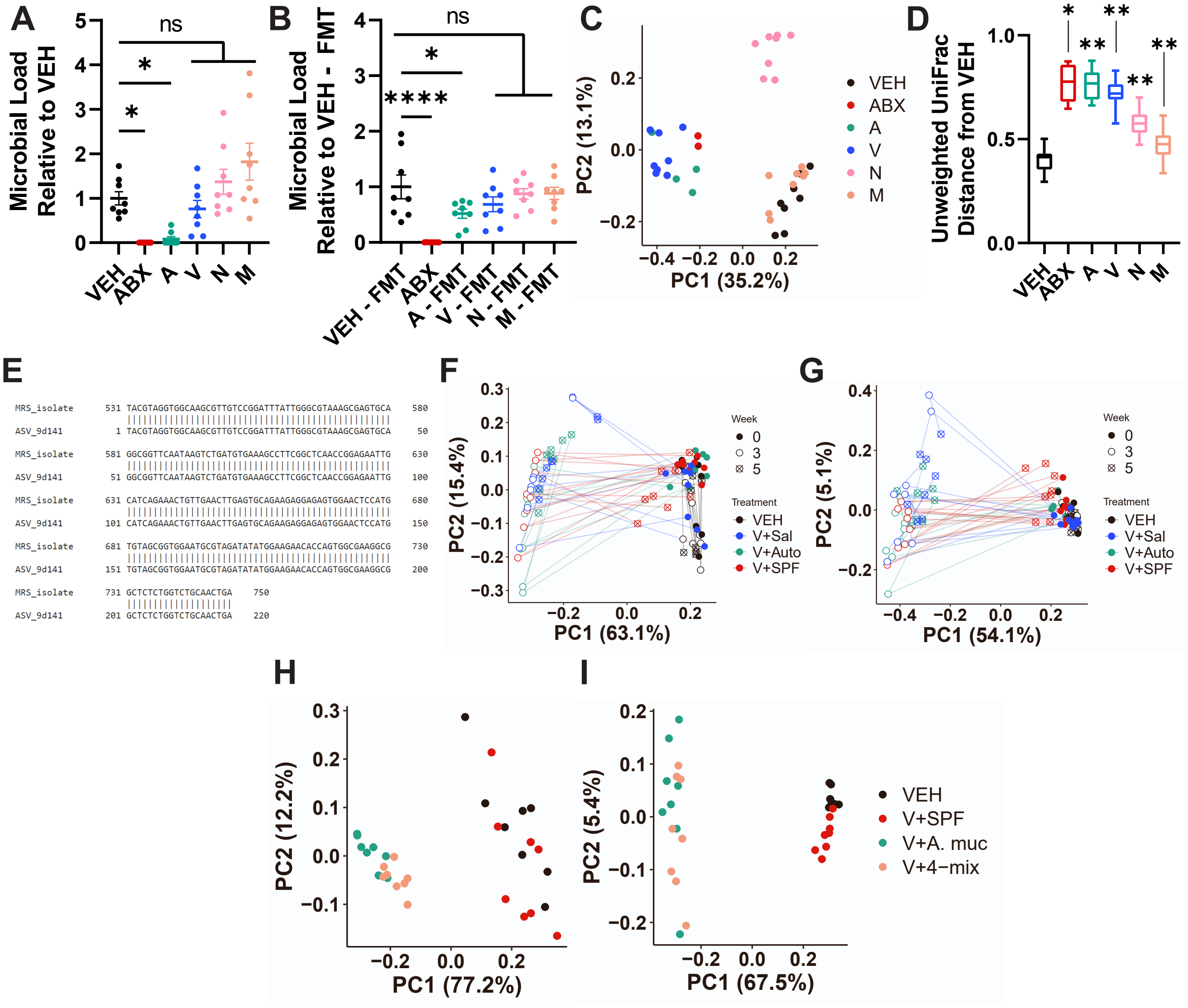

A
Microbial Load Relative to VEH
ns
*
*
VEH
ABX
A
V
N
M
B
Microbial Load Relative to VEH - FMT
ns
*
****
VEH - FMT
ABX
A - FMT
V - FMT
N - FMT
M - FMT
C
PC2 (13.1%)
PC1 (35.2%)
VEH
ABX
A
V
N
M
D
Unweighted UniFrac Distance from VEH
*
**
**
**
**
VEH
ABX
A
V
N
M
E
MRS_isolate 531 TACGTAGGTGGCAAGCGTTGTCCGGATTTATTGGGCGTAAAGCGAGTGCA 580
ASV_9d141 1 TACGTAGGTGGCAAGCGTTGTCCGGATTTATTGGGCGTAAAGCGAGTGCA 50
MRS_isolate 581 GGCGGTTCAATAAGTCTGATGTGAAAGCCTTCGGCTCAACCGGAGAATTG 630
ASV_9d141 51 GGCGGTTCAATAAGTCTGATGTGAAAGCCTTCGGCTCAACCGGAGAATTG 100
MRS_isolate 631 CATCAGAAACTGTTGAACTTGAGTGCAGAAGAGGAGAGTGGAACTCCATG 680
ASV_9d141 101 CATCAGAAACTGTTGAACTTGAGTGCAGAAGAGGAGAGTGGAACTCCATG 150
MRS_isolate 681 TGTAGCGGTGGAATGCGTAGATATATGGAAGAACACCAGTGGCGAAGGCG 730
ASV_9d141 151 TGTAGCGGTGGAATGCGTAGATATATGGAAGAACACCAGTGGCGAAGGCG 200
MRS_isolate 731 GCTCTCTGGTCTGCAACTGA 750
ASV_9d141 201 GCTCTCTGGTCTGCAACTGA 220
F
PC2 (15.4%)
PC1 (63.1%)
Week
0
3
5
Treatment
VEH
V+Sal
V+Auto
V+SPF
G
PC2 (5.1%)
PC1 (54.1%)
Week
0
3
5
Treatment
VEH
V+Sal
V+Auto
V+SPF
H
PC2 (12.2%)
PC1 (77.2%)
I
PC2 (5.4%)
PC1 (67.5%)
VEH
V+SPF
V+A. muc
V+4−mix

Figure S4. Differential antibiotic administration and microbial treatment has distinct effects on microbial abundance and diversity, Related to Figures 3 and 4

(A) Microbial load in fecal samples relative to VEH mice (n=8/group) as calculated by qPCR with universal bacterial primers. Shown is the mean (± SEM). Significance calculated via one-way ANOVA followed by Dunnett's multiple comparisons test to VEH.

(B) Microbial load in fecal samples relative to VEH - FMT mice (n=8/group) as calculated by qPCR with universal bacterial primers. Shown is the mean (± SEM). Significance calculated via one-way ANOVA followed by Dunnett's multiple comparisons test to VEH - FMT.

(C–D) PCoA and boxplot of pairwise comparisons of unweighted UniFrac distances in VEH (n=8), ABX (n=2), A (n=4), V (n=8), N (n=8), M (n=8) animals. Significance calculated by PERMANOVA. Asterisks in (D) denote significance of the PERMANOVA test of treatment groups compared to VEH. See also Table S1.

(E) EMBOSS matcher alignment of the 16S rRNA DNA sequence of a *L. johnsonii* isolate cultured from SPF feces (top) to the significantly differentially abundant ASV aligning to *Lactobacillus* sp. (bottom).

(F–G) PCoA of weighted (F) and unweighted (G) UniFrac distances of fecal microbiomes from vehicle (VEH) or vancomycin-pretreated mice that received treatment with saline vehicle (V+Sal), autologous FMT (V+Auto), or FMT from an SPF donor (V+SPF). Significance calculated via within-timepoint one-way ANOVA Kruskal-Wallis of within-subject distance comparisons to baseline followed by Mann-Whitney U tests subject to FDR correction as implemented in QIIME2 (n=8/group). See also Table S1.

(H–I) PCoA of weighted (H) and unweighted (I) UniFrac distances of fecal microbiomes from VEH, V+SPF, V+*A. muc*, and V+4-mix (n=8/group) mice. Significance calculated via PERMANOVA. See also Table S1.

Gene	Forward/Reverse	Source	Sequence (5’-3’)
AgRP	Forward	S1	TGCTACTGCCGCTTCTTCAA
AgRP	Reverse	S1	CTTTGCCCAAACAACATCCA
NPY	Forward	S1	TAACAAGCGAATGGGGCTGT
NPY	Reverse	S1	ATCTGGCCATGTCCTCTGCT
POMC	Forward	S1	AGGCCTGACACGTGGAAGAT
POMC	Reverse	S1	AGGCACCAGCTCCACACAT
18S *rRNA*	Forward	S2	TTCCGATAACGAACGAGACTCT
18S *rRNA*	Reverse	S2	TGGCTGAACGCCACTTGTC
16S 515F	Forward	S3	GTGCCAGCMGCCGCGGTAA
16S 806R	Reverse	S3	GGACTACHVGGGTWTCTAAT
16S 27F	Forward	S4	AGAGTTTGATCMTGGCTCAG
16S 1492R	Reverse	S5	GGTTACCTTGTTACGACTT

Table S3. Oligonucleotides used in this study, Related to STAR Methods

Supplemental References

S1. Piper, M.L., Unger, E.K., Myers, M.G., and Xu, A.W. (2008). Specific Physiological Roles for Signal Transducer and Activator of Transcription 3 in Leptin Receptor-Expressing Neurons. Mol. Endocrinol. *22*, 751–759. 10.1210/me.2007-0389.

S2. Reichenbach, A., Mequinion, M., Bayliss, J.A., Lockie, S.H., Lemus, M.B., Mynatt, R.L., Stark, R., and Andrews, Z.B. (2018). Carnitine Acetyltransferase in AgRP Neurons Is Required for the Homeostatic Adaptation to Restricted Feeding in Male Mice. Endocrinology *159*, 2473–2483. 10.1210/en.2018-00131.

S3. Caporaso, J.G., Lauber, C.L., Walters, W.A., Berg-Lyons, D., Lozupone, C.A., Turnbaugh, P.J., Fierer, N., and Knight, R. (2011). Global patterns of 16S rRNA diversity at a depth of millions of sequences per sample. Proc. Natl. Acad. Sci. *108*, 4516–4522. 10.1073/pnas.1000080107.

S4. Lane, D.J. (1991). 16S/23S rRNA sequencing. In Nucleic acid techniques in bacterial systematics., E. Stackebrandt and M. Goodfellow, eds. (John Wiley and Sons), pp. 115–175.

S5. Turner, S., Pryer, K.M., Miao, V.P., and Palmer, J.D. (1999). Investigating deep phylogenetic relationships among cyanobacteria and plastids by small subunit rRNA sequence analysis. J. Eukaryot. Microbiol. *46*, 327–338. 10.1111/j.1550-7408.1999.tb04612.x.
